# Genome-wide transcriptome study using deep RNA sequencing for myocardial infarction and coronary artery calcification

**DOI:** 10.1186/s12920-020-00838-2

**Published:** 2021-02-10

**Authors:** Xiaoling Zhang, Jeroen G. J. van Rooij, Yoshiyuki Wakabayashi, Shih-Jen Hwang, Yanqin Yang, Mohsen Ghanbari, Daniel Bos, BIOS Consortium, Daniel Levy, Andrew D. Johnson, Joyce B. J. van Meurs, Maryam Kavousi, Jun Zhu, Christopher J. O’Donnell

**Affiliations:** 1 Division of Intramural Research, National Heart, Lung and Blood Institute, Bethesda, MD USA; 2 The National Heart, Lung and Blood Institute’s Framingham Heart Study, Framingham, MA USA; 3 Department of Medicine (Biomedical Genetics), Boston University School of Medicine, 72 East Concord Street, Boston, MA 02118-2526 USA; 4 Department of Biostatistics, Boston University School of Public Health, Boston, MA USA; 5 Department of Internal Medicine, Erasmus Medical Center, Rotterdam, the Netherlands; 6 DNA Sequencing and Genomics Core, National Heart, Lung and Blood Institute, Bethesda, MD USA; 7 Department of Epidemiology, Erasmus Medical Center, Rotterdam, the Netherlands; 8 Department of Radiology and Nuclear Medicine, Erasmus Medical Center, Rotterdam, the Netherlands; 9 Cardiology Section, Veteran’s Administration Boston Healthcare System, Boston, USA

**Keywords:** Gene expression signatures, Protein-coding gene, Long intergenic non-coding RNA, Myocardial infarction, Coronary artery calcification, Whole blood, RNA-Seq

## Abstract

**Background:**

Coronary artery calcification (CAC) is a noninvasive measure of coronary atherosclerosis, the proximal pathophysiology underlying most cases of myocardial infarction (MI). We sought to identify expression signatures of early MI and subclinical atherosclerosis in the Framingham Heart Study (FHS). In this study, we conducted paired-end RNA sequencing on whole blood collected from 198 FHS participants (55 with a history of early MI, 72 with high CAC without prior MI, and 71 controls free of elevated CAC levels or history of MI). We applied DESeq2 to identify coding-genes and long intergenic noncoding RNAs (lincRNAs) differentially expressed in MI and high CAC, respectively, compared with the control.

**Results:**

On average, 150 million paired-end reads were obtained for each sample. At the false discovery rate (FDR) < 0.1, we found 68 coding genes and 2 lincRNAs that were differentially expressed in early MI versus controls. Among them, 60 coding genes were detectable and thus tested in an independent RNA-Seq data of 807 individuals from the Rotterdam Study, and 8 genes were supported by *p* value and direction of the effect. Immune response, lipid metabolic process, and interferon regulatory factor were enriched in these 68 genes. By contrast, only 3 coding genes and 1 lincRNA were differentially expressed in high CAC versus controls. *APOD*, encoding a component of high-density lipoprotein, was significantly downregulated in both early MI (FDR = 0.007) and high CAC (FDR = 0.01) compared with controls.

**Conclusions:**

We identified transcriptomic signatures of early MI that include differentially expressed protein-coding genes and lincRNAs, suggesting important roles for protein-coding genes and lincRNAs in the pathogenesis of MI.

## Highlights

- More than 25% long intergenic noncoding RNAs (lincRNAs) are detectable whole blood via deep RNA Sequencing with 150 million paired-end reads obtained for each sample on average.
- 68 protein-coding genes and 2 lincRNAs that were differentially expressed in early myocardial infarction (MI) cases versus controls.
- Immune response, lipid metabolic process, and interferon regulatory factor were enriched in these 68 protein-coding genes.
- Alternatively, only 3 coding genes and 1 lincRNA were differentially expressed in high coronary artery calcification (CAC) cases versus controls.
- *APOD*, encoding a component of high density lipoprotein, was significantly downregulated in both early MI and high CAC compared with the control group after adjusting for sex and 9 clinical vascular-related covariates, suggesting a potential novel target for the treatment and prevention of atherosclerotic disease.

## Background

Myocardial infarction (MI) is a leading cause of death in men and women worldwide [1, 2] Genetic inheritance is a major component to MI risk, particularly for early onset MI [1]. Coronary artery calcification (CAC) is directly correlated with quantity of coronary atherosclerotic plaque [3]. CAC detected by computed tomography is a noninvasive measure of coronary atherosclerosis and a CAC score is a strong independent predictor of future MI [4] including early MI [5, 6]. Genome-wide association studies (GWAS) have identified common and rare genetic variants associated with both CAC and early MI, including variants in the 9p21, *SORT1* and *PHACTR1* loci [7–11]. However, the molecular mechanisms underlying early MI and CAC remain unclear. In particular, data are sparse regarding gene expression signatures for early MI and for subclinical coronary atherosclerosis, detected as high CAC.

RNA sequencing for atherothrombotic cardiovascular disease offers a complementary genome-wide molecular approach to investigate disease-related mechanisms by measuring expression abundance of protein-coding genes (mRNA) and long intergenic non-coding genes (lincRNAs) in specific tissues. Altered expression levels in disease can reflect the effects of genetic variation, environmental effects, interaction between genetic variation and environmental effects, and the effects of the disease process itself or drugs used for its treatment. We conducted deep paired-end RNA sequencing (RNA-Seq) on whole blood samples collected from Framingham Heart Study (FHS) participants. Blood is an easily accessible tissue relevant for expression profiling of cardiovascular disease and its risk factors, has the advantage of providing information on patients’ real-life state in contrast with cell-lines, and can be extended to very large sample sizes for biomarker screening.

Our current study aimed to generate and characterize coding and noncoding gene expression signatures of early-onset MI and CAC in whole blood collected from a single large cohort, the Framingham Heart Study (FHS), and to further examine the relationships between high CAC and early-onset MI based on expression profiling of whole blood. We studied 198 European ancestry individuals (55 with history of early MI, 72 with high CAC without MI, and 71 control participants free of elevated CAC levels or history of MI) with whole-blood RNA-Seq. To the best of our knowledge, this is a first whole-transcriptome study using RNA-Seq in participants with a history of prior MI or coronary atherosclerosis detected by the presence of CAC in a single study sample. We first performed a genome-wide transcriptome screen to identify blood-specific transcripts including mRNA and lincRNAs. Second, we conducted association analyses between MI/CAC and the expression levels of individual mRNAs and of lincRNAs. Last, we categorized the functional pathways of differentially expressed genes (DEGs) between MI/CAC and controls to identify biological functions of the differentially expressed genes. Using deep coverage RNA-Seq data, we identified 12,062 protein-coding genes and 3707 lincRNAs expressed at relatively high levels in blood as well as significant MI-specific expression signatures, with eight genes (15%) supported in an independent cohort with RNA-Seq data. We sought to provide insights into mechanisms through which transcriptome-level variation may influence the development of subclinical coronary atherosclerosis and, ultimately, clinical MI.

## Methods

### Study population and sample collection

The FHS started in 1948 with 5209 randomly ascertained participants from Framingham, MA, who underwent biennial examinations to investigate cardiovascular disease and its risk factors [12]. In 1971, the Offspring cohort [13, 14] (comprised of 5124 children of the original cohort and the children’s spouses) and in 2002, the Third Generation (consisting of 4095 children of the Offspring cohort), were recruited [15]. Participants of the Framingham Heart Study (FHS) Offspring cohort who attended examination 8 (*n* = 202, 57 with early MI, 74 with high CAC without MI, and 71 control participants free of elevated CAC levels and MI matched with age and sex) were included, constituting a total of 198 individuals. The clinical characteristics of the FHS Offspring participants included in this study are presented in Table 1, and they are European ancestry. The study protocol was reviewed by the Boston University Medical Center Institutional Review Board, and all participants gave written informed consent.

**Table 1:** Clinical characteristics of the FHS Offspring participants included in this study (*N* = 198) at examination 8

*N* = 198	Early MI (*n* = 55)	High CAC w/o MI (*n* = 72)	Controls (*n* = 71)	*P*-value
Age (years)	68.47 ± 7.80	67.73 ± 9.19	67.46 ± 5.78	0.76
Sex	42 M, 13F	30 M, 42F	36 M, 35F	0.00037
BMI (kg/m^2^)	29.07 ± 4.72	29.39 ± 5.60	28.99 ± 5.46	0.937
SBP (mmHg)	125.3 ± 17.62	132 ± 17.01	127.3 ± 15.06	0.064
DBP (mmHg)	69.02 ± 9.24	73.58 ± 10.49	74.49 ± 8.71	0.0041
TC (mg/dl)	154.7 ± 33.79	179.9 ± 34.22	183.2 ± 31.97	4.49E-06
TC_HDL	3.38 ± 1.12	3.62 ± 1.15	3.42 ± 0.97	0.41
Triglycerides (mg/dl)	126.4 ± 82.09	138.1 ± 87.1	114.4 ± 53.14	0.17
HDL (mg/dl)	48.69 + 14.53	53.06 + 13.94	57.58 (18.83)	0.009
Fasting glucose (mg/dl)	111.2 ± 30.76	112.5 ± 26.63	105 ± 14.85	0.16
Hypertension Rx (%)	54	47	37	1.02E-07
Diabetes Rx (%)	13	18	7	0.042
Lipid Rx (%)	51	46	32	1.78E-07
Cigarette use (current) (%)	9	5	2	0.02
Aspirin Rx (%)	44	43	31	0.0002
Diabetes mellitus (%)	11	15	9	0.04

*MI* Myocardial infarction, *CAC* Coronary artery calcification, *BMI* Body mass index, *SBP* Systolic blood pressure, *DBP* Diastolic blood pressure, *TC* Total cholesterol, *TC_HDL* TC/HDL, *HDL* High-density lipoprotein cholesterol, *Rx* drug therapy

### RNA library preparation, sequencing, and data processing

Fasting peripheral whole blood samples (2.5 ml) were collected in PAXgene™ tubes (PreAnalytiX, Hombrechtikon, Switzerland), incubated at room temperature for 4 h for RNA stabilization, and then stored at − 80 °C. Total RNA was isolated from frozen PAXgene blood tubes by Asuragen, Inc., according to the company’s standard operating procedures for automated isolation of RNA from 96 samples in a single batch on a KingFisher® 96 robot. RNA was extracted; most globin RNA was removed (GLOBINclear Kits, Life Technologies, and Grand Island, NY, USA). 10 ng of total RNA was used as input for RNA-Seq library construction using Ovation RNAseq v2 (NuGEN Technologies, Inc., San Carlos, CA), following the guidelines for the Ovation SP Ultralow Multiplex System (NuGEN Technologies, Inc., San Carlos, CA). After the final amplification step, libraries were size selected between 250 to 450 base pairs. Library quality was verified for each sample using MiSeq (Illumina, Inc., San Diego, CA), sequencing with 75-bp paired-end reads. Sequencing data production was carried out with Illumina HiSeq 2000 (Illumina, Inc.; 75-bp pair-ended reads, 1 library/sample per lane) for 202 individuals, yielding 150 million paired-end reads (average) per individual. Reads were mapped to the NCBI v37 *Homo sapiens* reference genome using Tophat. Complete RNA-seq data are available in dbGaP (see data access note below). Details of RNA isolation, preparation of cDNA from RNA, and RNA-sequencing and data processing are available in the Additional file 1: Supplemental Material.

**Fig. 1:**
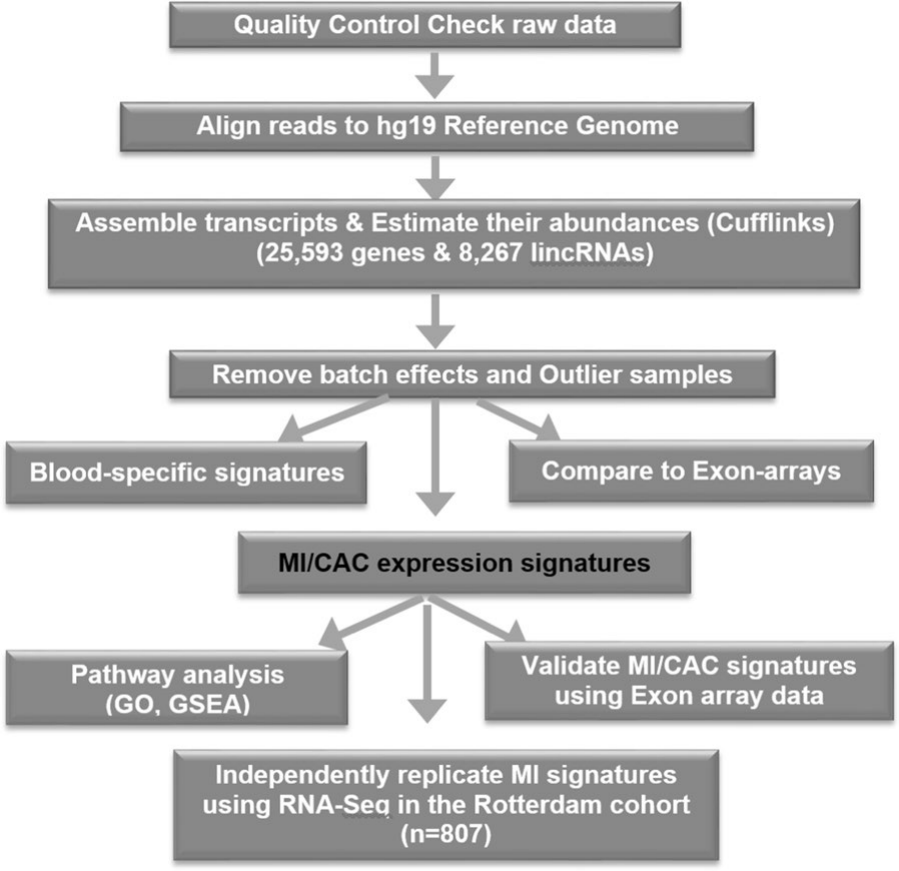
Flowchart of sequencing mapping, quantification and expression analysis of RNA-Seq data to identify coding and non-coding expression signatures associated with MI and CAC

Data quality of raw sequencing data (.fastq) for each sample is assessed using FASTQC, and 75 bp paired-reads are aligned to the human reference genome sequence (hg19) using Bowtie2 within Tophat2 [16]. Samples with a low overall mapping rate (less than 50%) are defined as outliers and excluded from downstream analysis. We also sequenced 9 samples twice. For samples sequenced twice, the sample with higher number of sequenced reads and unique mapping rate was retained in the analysis. After assessment of quality based on mapping rate, sex mismatch checking using gene markers on chromosome Y, and outlier detection by principle component analysis (PCA), a total of 198 samples remained for use in all downstream analysis of this study. The flowchart of this analysis pipeline is shown in Fig. 1**.**

### Characterization of coding genes and non-coding lincRNAs that are specifically expressed in blood using deep RNA-Seq

After alignment, using an annotation file (Ensembl) [17], fragments per kilobase of transcript per million mapped reads (FPKM) values are derived using Cufflinks [18] to be used as expression measurements for each feature (22,881 protein-coding genes and 7364 lincRNAs). For Illumina BodyMap RNA-seq data which contains 16 human tissues, the raw .fastq files were downloaded and processed as described above to obtain FPKM values for each transcript. After normalizing data and removing batch effects [19], a linear regression model is applied to identify coding genes and lincRNAs that are expressed at a higher level in blood than in 16 other tissues in the BodyMap. Candidate genes are viewed in IGV [20, 21]. Expression measurements for coding-genes and lincRNAs were quantified by using Cufflinks as reported recently [22, 23].

### Estimation of hidden confounders and their association with known clinical phenotypes and technical variables

Besides known batch effects (sequencing batch) and known covariates, in order to take into account known and unknown technical effects, and other unwanted variations (e.g., proportions/frequencies of the various cell types present in whole blood) in the analysis, we applied the Surrogate Variable Analysis (SVA) [24] to calculate hidden variables, but in the meanwhile preserving biological heterogeneity in high-throughput experiments. These computed hidden variations are needed to be adjusted in the downstream analysis model to decrease false positives.

FPKM values of all transcripts estimated (including coding genes, lincRNAs, antisense transcripts, pseudogenes, etc) were used as the input for SVA package to estimate the surrogate variable (SV). Two significant SVs were finally selected and included in the downstream association analysis.

### Identification of coding-gene and lincRNA expression signatures associated with MI and CAC

For our differential expression analysis, we first filtered for stably expressed mRNAs and lincRNAs in whole blood (FPKM > 0.1 in > = 10% of samples). After filtering non-expressed genes, for each of 12,062 protein-coding genes and 3707 lincRNAs, we applied DESeq2 [25] to identify genes differentially expressed in MI and high CAC, respectively, compared with the control group. Since sex are statistically different among early MI, high CAC and control (*P* = 0.00037), we adjusted for sex besides known batch effects and hidden confounders (i.e. 2 SVs estimated from the above step) in the model in which the raw count is the outcome (dependent variable), disease status (MI/CAC/control) is the predictor, and age, known batch effects and hidden confounders were included as covariates. Since these 198 participants were selected from un-related families, no family structure was adjusted for in the model. We applied the package DESeq2 in R to estimate the disease effects**.**

#### Protein-coding gene expression association analysis

A total of 12,062 tests were performed examining the associations between each of the expressed coding-genes and MI and high CAC, respectively, compared with the control group. Following a Bonferroni multiple test correction (Benjamini and Hochberg method), a false discovery rate (FDR) < 0.1 (corresponding to a nominal *P* value of 5.61E-4) was used to define significant associations between the expression level of code-genes and the disease status (differentially expressed coding genes with MI and high CAC, respectively). In addition, for genes differentially expressed between MI and control at FDR < 0.1, we performed a secondary, hypothesis-generating analysis to test expression level changes between MI and high CAC.

#### LincRNA expression association analysis

A total of 3707 tests were performed examining the associations between each of the expressed lincRNAs and MI and high CAC, respectively, compared with the control group. The same significance level of FDR < 0.1 (corresponding to a nominal *P* value of 6.33E-05) was used to define significant associations between the lincRNA expression level and the disease status (differentially expressed coding genes with MI and high CAC, respectively).

### Gene ontology enrichment analysis to examine biological functions of gene signatures

For the 435 coding genes expressed moderately and highly in blood, we submitted their unique gene symbol to the DAVID website [26, 27] to identify GO molecular function categories, KEGG pathways, and CGAP BioCarta Pathways over-represented among the 435 coding genes compared to background (all human genes). We accounted for multiple testing using Bonferroni-corrected significance levels: 0.05/1472 = 0.00003 (cellular component) or 0.05/8972 = 0.000006 (biological process). The same analysis was conducted for the 68 coding genes differentially expressed between MI and control participants (FDR < 0.1).

### Gene set enrichment analysis (GSEA)

The Molecular Signatures Database (MSigDB) is a collection of annotated gene sets for use with GSEA software [28]. Gene sets from the MSigDB were used to search for pathways that may be more modestly altered in MI-associated gene expression data. A ranked gene list was generated by ranking t-value of 12,062 filtered genes in differentially expressed gene analysis using DeSeq2 with RNA-Seq count data. Then a total of 3283 gene sets in GSEA C2 category [28] (MSigDB) were used for the enrichment analysis. FDR < 0.25 was used to define the significance of enrichment. Gene set size of < 15 and > 500 were filtered out, resulting in filtering out 1442/4725 gene sets. Therefore, the remaining 3283 gene sets were used in the analysis.

### Comparison of RNA-Seq and exon array platform and validation of MI gene signatures detected from deep RNA-Seq data using Affymetrix exon array data

In a sample of 198 samples with high quality RNA-Seq data being used in the analysis of this study, 193 RNA samples were also analyzed by Affymetrix Exon-array to obtain gene-level measurements. In order to be comparable between these two platforms, We first created a custom BED file based on the coordinates of core probe sets on exon-arrays so that only reads mapping to core-probe sets are used by RSeQC [29] to obtain gene-level RPKM values. We found that the overall correlation of coding genes between RNA-Seq and Exon-array is only a little lower (*r*^^2^ = 0.56, Pearson correlation coefficient *r* = 0.75) than a previous study (*r*^^2^ = 0.62) [30], indicating the high quality of RNA-Seq human blood data considering that the previous study [30] was conducted in cell line samples.

We have identified 68 coding genes differentially expressed between 55 early MI and 71 control participants in whole blood, and then these genes were classified as MI genes. In order to estimate the relationship of the MI genes between RNA-Seq data in this study and Exon array data obtained before, MI genes were mapped to Exon array dataset via gene symbol. PCAs were performed for each dataset across the mapped genes using z-score normalized data. The relationship was subsequently defined quantitatively using GSEA. The samples in each dataset were divided into two groups: case versus control (MI vs. control). Then all of the genes in Exon array dataset were ranked by the signal to noise statistic (t value) in the MI case: control comparison. GSEA was used to determine if the gene set (MI-associated genes) detected from deep RNA-Seq data were significantly enriched in the above ranked gene list generated from Exon array data.

### Replication of MI expression signatures using IlIumina RNA-Seq in an independent Rotterdam cohort

Rotterdam Study RNA-Seq data was generated as part of the BIOS project and is described in detail elsewhere [31]. In short, total RNA was globin cleared using Ambion GLOBINclear and sequenced to a minimum yield of 15 M paired-end reads on HiSeq 2000. Data was aligned to reference genome hg19 using STAR, followed by quantification of all GENCODE v16 genes using custom scripts. We then extracted the counts of 56,515 transcripts for 807 participants of the Rotterdam Study (RS-I, II and III). Using edgeR, counts per million (CPM) mapped reads were generated for each sample, and transcripts with CPM < 1 in more than 90% of samples were excluded, allowing 15,331 transcripts in further analysis. In summary, among 404 individuals (15 from RS-I and 389 from RS-II, 190 male and 214 female), there are 28 MI cases vs. 376 controls and 41 CHD cases vs. 363 controls for the differential expression analyses.

For each coded phenotype (MI/CHD), linear regression analysis was performed in R, correcting for age, gender, flow-cell and the number of sequenced reads. A custom linear regression script was used as the dataset was too large to be processed by edgeR or DESeq. The effect sizes and uncorrected *p*-values were reported for each of the candidate genes of the discovery analysis.

See Additional file 1: Supplemental Material for details of Rotterdam cohort and its measurement of CAC.

## Results

### Sample characteristics and RNA quality of the blood samples

Two hundred two FHS Offspring participants with a history of early MI, high CAC, or neither and with whole blood expression data were selected for this RNA sequencing study. After exclusion for poor quality and outliers of two participants with history of early MI and two participants with high CAC, 198 samples remained for inclusion in the analysis. Our final study sample with RNA-Seq of whole-blood RNA consisted of 198 European ancestry individuals (55 with history of early MI, 72 with high CAC without MI, and 71 control participants free of elevated CAC levels or history of MI). Clinical characteristics of these study participants are presented in Table 1. Participant selection was targeted to match for age and sex across three groups. As shown in Table 1, there was no difference in age (mean = 68, *P* = 0.76), but there were differences in the distributions of sex (*P* = 0.00037) and several other clinical covariates across groups including diastolic blood pressure, total cholesterol, HDL-C, smoking, diabetes and treatments for hypertension, dyslipidemia, or diabetes as well as use of aspirin (*P* < 0.05).

We compared the RNA quality across the three groups. The concentration and yield of RNA were slightly different (*P* = 0.03) with a relatively higher concentration and yield in controls, respectively. However, as shown in Additional file 1: Table S1, there are no differences among groups for RNA quality score i.e., RNA integrity number (RIN), and 260/280 ratio.

### Transcriptome profiling of coding and non-coding genes in human whole blood

By sequencing one sample per lane using Illumina HiSeq platform, we generated high-quality and deep coverage RNA-Seq data for 201 blood samples besides nine replicates. On average, there were 150 million paired-end 75 bp reads for each sample. The overall unique mapping rate is 75% and the concordant pair alignment rate was 62%. Sequencing summary and mapping statistics are shown in Additional file 1: Fig. S1. For the nine samples sequenced twice, we checked the correlation of all measured transcriptome in whole blood between samples sequenced twice, which ranged from 0.77 to 0.99 (Additional file 1: Table S2). After QC, in the final analysis, for samples sequenced twice, we selected the one with the higher number of total unique mapped reads. Samples were also checked for sex mismatch. After exclusion for poor quality RNA and outliers, 198 samples remained for inclusion in the analysis.

Of 22,881 Ensembl protein-coding genes, 56% (12,823/22,881) were detectable (log_2_[FPKM] > 1 in > 10% of the samples, FPKM = fragments per kilobase of transcript per million mapped reads). For protein-coding genes expressed in all samples, we found 4133 genes with log_2_[FPKM] > 1, and 435 genes with log_2_[FPKM] > 4. Gene ontology analysis showed that categories of immune and defense response, leukocyte activation, calcium binding, leukocyte migration and adhesion were enriched in the 435 genes expressed moderately and highly in blood (Additional file 1: Table S4). Using a cutoff of log_2_[FPKM] > 0.1, 25.6% (1886/7364) lincRNAs are detectable in > 10% of the samples. For lincRNAs expressed in all samples, we found only 36 lincRNAs with log_2_[FPKM] > 1, and 2 with log_2_ [FPKM] > 4.These findings are consistent with prior observations that the expression levels of lincRNAs are much lower than those of mRNAs [32]. All protein-coding genes expressed at > = 4 log_2_[FPKM] and all lincRNAs expressed at > = 1 log_2_[FPKM] in all samples are listed in Additional file 1: Tables S3 and S5 in the online-only Data Supplement, respectively.

We further identified protein-coding genes and lincRNAs that were more highly expressed in whole blood by comparing to expression in other tissues in the Illumina Human Body Map RNA-Seq data. The Illumina Human BodyMap 2.0 data [33] includes RNA-Seq data for 16 other human tissues, and we processed the Illumina Human BodyMap 2.0 data with the same tools and parameters as for our blood RNA-Seq data. In this comparison, ~ 60% of the detectable coding-genes were expressed much higher in our blood samples than in 16 other human tissues. As shown in the heat map of 52 genes highly expressed in blood (log_2_[FPKM] > 6 in 100% samples) and 36 lincRNAs moderately expressed in blood (log_2_[FPKM] > 1 in 100% samples), half of coding genes are expressed specifically in blood (Additional file 1: Fig. S2a), and two thirds of lincRNAs are expressed higher in blood compared to other 16 human tissues. By evaluating the expression profiles of 36 lincRNAs expressed highly in blood (log_2_ [FPKM) > 1 in all samples), two clusters were identified as shown in Additional file 1: Fig. S2b. Among them, the expression level of 26 lincRNAs (top panel) is substantially higher in blood compared to 16 other human tissues in the Human BodyMap data, including the FAM157A lincRNA, a known lincRNA known to be overexpressed in blood, that contains 14 exons and has 2 transcripts (splice variants) as shown in the Ensembl Genome Browser [34].

Finally, we compared the overall expression profiling on Illumina RNA-Seq and Affymetrix Exon-array platforms in all 193 participants with samples with expression data from both platforms. The correlation of expression level between the two platforms is on average 0.745 with a median of 0.748, which is slighter lower (*R*^^2^ = 0.56) than a prior report with an R^^2^ of 0.62 between RNA-Seq and Exon-array data for 5 cell line samples [30]. The correlation result is high, with overall *r* = 0.75. Additional file 1: Fig. S3a shows the correlation plot for one sample, and Additional file 1: Fig. S3b shows the distribution of r values for all 193 samples.

### Differential expression analysis to identify mRNA and lincRNA signatures for early MI and high CAC

At a false discovery rate (FDR) < 0.1, we found 68 coding genes and two lincRNAs differentially expressed between early MI and controls (Table 2**),** with 21 genes overexpressed in MI cases and 49 down-regulated, including two lincRNAs. By contrast, only three coding genes and one lincRNA (RP11-245 J9) were differentially expressed between high CAC and controls (Table 3**)**. Notably, *APOD*, encoding a component of high-density lipoprotein, was expressed significantly lower in both early MI (FDR = 0.007) and in high CAC (FDR = 0.01) compared with controls, respectively, highlighting a novel candidate for both MI and subclinical atherosclerosis.

**Table 2:** Top MI-associated genes (68 protein-coding and 2 lincRNAs) at FDR < 0.1. Eight coding genes supported by *p* value and direction are in bold

		Discovery (55 MI cases)				Replication (28 MI cases, 41 CHD cases)		
Gene Symbol	locus	Base Mean	log2Fold Change	Raw_***p*** value	FDR	Rep_MI_***p*** value	Rep_MI_direction	Rep_CHD_***p*** value
*APOD*^*a*^	3:195295572-195,311,076	301	− 0.19	1.29E-06	0.0075	0.63	Yes	0.51
*DUS1L*	17:80015381-80,023,763	902	0.41	1.97E-06	0.0075	0.778	No	0.331
*AFF3*	2:100162322-100,759,201	1478	−0.44	2.12E-06	0.0075	0.326	Yes	0.397
*IRF7*	11:612552-615,999	1652	0.41	2.50E-06	0.0075	0.286	Yes	0.378
**IPO5**	**13:98605911-98,676,551**	**1866**	**−0.32**	**3.75E-06**	**0.0090**	**0.0416**	**Yes**	0.115
*SH3PXD2A*	10:105348284-105,615,301	927	−0.40	5.93E-06	0.0119	0.248	Yes	0.0541
*BACH2*	6:90636247-91,006,627	3905	−0.37	1.09E-05	0.0188	0.48	Yes	0.586
*PAX5*	9:36833271-37,034,103	1406	−0.41	1.67E-05	0.0252	0.451	Yes	0.339
*PHF6*	X:133507282-133,562,820	704	−0.33	1.99E-05	0.0267	0.523	Yes	0.837
*FCRL2*	1:157715522-157,746,922	868	−0.40	2.26E-05	0.0272	0.482	Yes	0.37
*VEZT*	12:95611521-95,696,566	1024	−0.32	2.97E-05	0.0301	0.674	Yes	0.896
*TRIM46*	1:155145872-155,157,447	159	0.39	3.52E-05	0.0301	0.429	Yes	0.22
**IFI6**	**1:27992571-27,998,729**	**1711**	**0.39**	**3.60E-05**	**0.0301**	**0.0291**	**Yes**	0.0658
*RAD52*	12:1021242-1,099,219	1090	−0.30	3.75E-05	0.0301	0.65	No	0.394
*BLK*	8:11351509-11,422,113	562	−0.39	3.87E-05	0.0301	0.168	Yes	0.125
*HLA-F*	6:29690551-29,706,305	5562	0.27	3.99E-05	0.0301	0.307	Yes	0.286
*IFI27*	14:94571181-94,583,033	170	0.32	4.54E-05	0.0306	0.506	Yes	0.783
**HNRNPR**	**1:23630263-23,670,829**	**2946**	**−0.26**	**4.56E-05**	**0.0306**	**0.00712**	**Yes**	**0.0141**
*ZNF44*	19:12335500-12,405,702	671	−0.30	5.16E-05	0.0319	0.704	No	0.336
*FCER2*	19:7753643-7,767,032	592	−0.38	5.29E-05	0.0319	0.904	Yes	0.747
*FRS2*	12:69864128-69,973,562	1778	−0.26	5.88E-05	0.0328	0.913	No	0.576
*HBG1*	11:5269312-5,271,122	17,515	0.35	5.98E-05	0.0328	0.0753	Yes	0.38
*PRKDC*	8:48685668-48,872,743	9155	−0.22	6.59E-05	0.0337	0.41	Yes	0.907
*MS4A1*	11:60223224-60,238,233	3170	−0.38	6.91E-05	0.0337	0.217	Yes	0.163
*FCRLA*	1:161676761-161,684,142	562	−0.37	6.98E-05	0.0337	0.187	Yes	0.112
*ACADVL*	17:7120443-7,128,592	3648	0.26	8.06E-05	0.0370	0.061	No	0.0195
*CCDC141*	2:179694483-179,914,813	703	−0.37	8.28E-05	0.0370	0.398	Yes	0.884
**HBG2**	**11:5274419-5,667,019**	**39,577**	**0.33**	**9.13E-05**	**0.0393**	**0.0082**	**Yes**	0.0885
*SKIL*	3:170075465-170,114,623	1570	−0.25	9.53E-05	0.0396	0.42	No	0.493
*GPT2*	16:46918289-46,965,209	100	−0.32	0.00012109	0.0487	0.414	Yes	0.267
*STRBP*	9:125871778-126,030,855	1034	−0.34	0.00013802	0.0537	0.213	Yes	0.161
*ZNF445*	3:44481261-44,519,162	2182	−0.22	0.00014638	0.0552	0.995	Yes	0.681
*RASGRF1*	15:79252288-79,383,115	84	−0.22	0.00017229	0.0630	NA		NA
*FIGNL1*	7:50511830-50,518,088	234	−0.35	0.00017999	0.0639	0.61	No	0.394
**PRKX**	**X:3522410-3,631,649**	**2556**	**−0.25**	**0.00018916**	**0.0652**	**0.0114**	**Yes**	0.123
*CLNK*^*a*^	4:10488018-10,686,489	96	−0.29	0.00020296	0.0670	NA		NA
*DDX6*	11:118620033-118,661,858	10,805	−0.18	0.0002167	0.0670	0.986	No	0.62
*DESI2*	1:244816236-244,872,335	945	−0.27	0.00021823	0.0670	0.24	Yes	0.564
**NPDC1**	**9:139933921-139,940,655**	**146**	**0.34**	**0.00021874**	**0.0670**	**0.000883**	**Yes**	**0.00258**
*NXF1*	11:62559594-62,573,774	7228	0.16	0.00022235	0.0670	0.864	Yes	0.973
*RNF113A*	X:119004496-119,005,791	297	0.34	0.00025995	0.0737	NA		NA
*RARS*	5:167913449-167,946,304	1359	−0.27	0.0002653	0.0737	0.496	Yes	0.401
*RHOBTB2*	8:22844929-22,877,712	558	−0.34	0.0002664	0.0737	0.0741	Yes	0.0351
*UGGT1*	2:128848773-128,953,251	4889	−0.18	0.00026872	0.0737	0.176	Yes	0.31
*ISG15*	1:948802-949,920	635	0.34	0.00028111	0.0752	0.382	Yes	0.635
*TIPARP*	3:156391023-156,424,559	938	−0.26	0.00028679	0.0752	0.434	Yes	0.789
*KSR2*	12:117890816-118,406,788	98	−0.15	0.00029511	0.0757	NA		NA
*ATP6V0D1*	16:67471916-67,515,140	8907	0.20	0.00032105	0.0807	0.792	Yes	0.966
*FBXO11*	2:48016454-48,132,932	1609	−0.23	0.00033474	0.0811	0.182	No	0.0272
*ZNF274*	19:58694395-58,724,928	840	−0.29	0.00033608	0.0811	0.668	No	0.196
*MCOLN1*	19:7587511-7,598,895	1777	0.32	0.00034818	0.0823	0.321	Yes	0.494
**DDX24**	**14:94517265-94,547,591**	**2085**	**−0.27**	**0.0003551**	**0.0824**	**0.0259**	**Yes**	0.138
*PEX26*	22:18560688-18,613,905	1308	−0.26	0.00036948	0.0835	0.0651	Yes	0.025
*TBC1D23*	3:99979843-100,044,095	1794	−0.22	0.00037386	0.0835	0.756	No	0.399
*WNT3*	17:44839871-44,910,520	137	−0.24	0.00039387	0.0858	NA		NA
*RAB30*	11:82684174-82,782,965	804	−0.31	0.00040083	0.0858	0.659	Yes	0.635
*ODF3B*	22:50968138-50,971,009	533	0.33	0.00040552	0.0858	0.36	Yes	0.265
*CDK2AP2*	11:67273967-67,276,120	771	0.32	0.00042475	0.0872	0.604	No	0.365
**CDKN2D**	**19:10677137-10,679,735**	**4162**	**0.28**	**0.00043108**	**0.0872**	**0.0279**	**Yes**	0.0662
*SLC6A16*	19:49792894-49,828,482	611	−0.32	0.00043371	0.0872	0.654	Yes	0.38
*MOAP1*	14:93648540-93,651,273	427	0.32	0.00044545	0.0881	0.0415	No	0.0489
*IRF4*	6:391738-411,447	1629	−0.30	0.00046627	0.0907	0.206	Yes	0.47
*S100PBP*	1:33282367-33,324,476	2679	−0.23	0.00048111	0.0913	0.918	No	0.843
*GPR15*	3:98250742-98,251,960	181	0.28	0.00048423	0.0913	0.162	Yes	0.0226
*DNAH7*	2:196602426-196,933,536	151	−0.33	0.00051455	0.0939	NA		NA
*TCF20*	22:42556018-42,739,622	4310	−0.21	0.00052057	0.0939	0.216	Yes	0.307
*EIF2S3L*	12:10658200-10,675,734	393	−0.27	0.00052155	0.0939	NA		NA
*PSMB6*	17:4699438-4,701,790	615	0.31	0.00056066	0.0995	0.406	No	0.245
*LINC00452*^*b*^	13:114586639-114,588,308	14	−1.33	1.44E-05	0.0453	NA		NA
*RP11-481 J2.2*^*b*^	16:58455229-58,496,374	10	−1.21	6.33E-05	0.0997	NA		NA

Significance of bold are those 8 coding genes replicated at *p* value

^a^Two genes (*APOD*, *CLNK*) are also associated with high CAC as shown in Table 3

^b^lincRNAs; *FDR* False Discovery Rate, *Rep* Replication, *MI* Myocardial Infarction, *CHD* Coronary Heart Disease, *NA* genes not found in the Replication Rotterdam Study cohort, *Base Mean* the average of reads mapped to this gene across all samples, *Rep_MI_direction* the effect direction (i.e., log2FoldChange direction) in our discovery cohort is the same as in the Replication cohort

**Table 3:** Top CAC-associated genes (3 protein-coding and 1 lincRNA) at FDR < 0.1

Gene Symbol	locus	Gene_biotype	Base Mean	log2Fold Change	*P*.value	FDR
*APOD*^*a*^	3:195295572-195,311,076	protein_coding	300.6917	−0.211	1.12E-06	0.0135
*CLNK*^*a*^	4:10488018-10,686,489	protein_coding	96.22257	−0.364	7.38E-06	0.039
*RASGEF1A*	10:43689982-43,762,367	protein_coding	208.1619	0.418	9.71E-06	0.039
*RP11-245 J9.5*^*b*^	3:63993757-63,994,368	lincRNA	435.893	−0.59136	4.94E-05	0.091

^a^Two genes are also associated with MI as shown in Table 2; ^b^lincRNAs; *FDR* False Discovery Rate

**Fig. 2:**
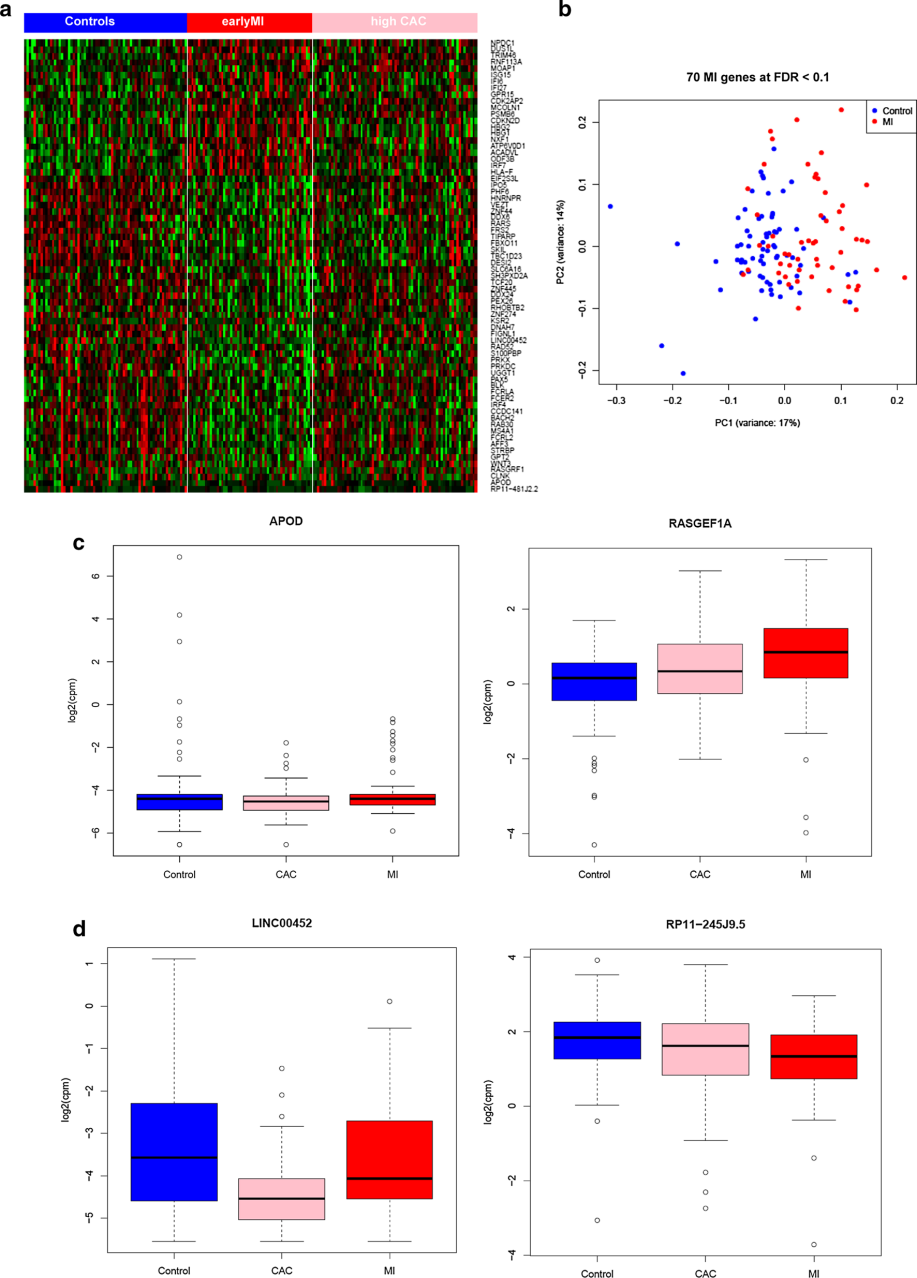
MI and CAC expression signatures. **a** Heatmap of 198 samples showing substantial differential expression changes across three groups (Controls, early MI, and high CAC). **b** Principle component analysis (PCA) of 70 MI gene signatures (68 protein-coding and 2 lincRNAs) shows a separation pattern between MI and controls. **c** Examples of the genes significantly associated with both MI and high CAC. The boxplot shows a low expression level in early MI and high CAC compared to controls for *APOD* and vice versa for *RASGEF1A*. **d** Boxplot of 2 lincRNAs that are moderately expressed in blood and associated with MI and CAC by RNA-Seq

A supervised clustering analysis of the 69 expression signatures (68 MI and 3 CAC genes with 2 genes shared by both MI and CAC) shows significant expression changes across the three groups (Fig. 2a). Principle component analysis (PCA) of 68 MI gene signatures (Fig. 2b) also shows a separation pattern between MI and controls. In addition, for DEGs, particularly for *APOD* detected in both MI and high CAC, boxplots show a clear trend in early MI and high CAC relative to controls (Fig. 2c). *APOD* is ranked as the top gene in both MI (log_2_ fold change = − 0.2, FDR = 0.007) and high CAC (FDR = 0.01) gene signatures. Of note, *APOD* was moderately expressed in blood with an average of FPKM of 3.79 across all 198 samples.

To assess whether our findings may represent transcriptomic signatures confounded by baseline clinical variables/covariates, established vascular risk factors, or of drug treatments for MI or its risk factors, we further adjusted for 9 covariates that differed among the three groups (see Table 1) in the primary simple differential analysis model in which we had adjusted for sex, known batch effects, and hidden confounders (i.e. two SVs). The 9 clinical covariates include diastolic blood pressure, total cholesterol, HDL-cholesterol, cigarette smoking, diabetes, and drug treatment for hypertension, dyslipidemia, or diabetes as well as use of aspirin. For all 70 MI genes reported in Table 2, after adjustment for these covariates, except for *APOD* and *DUS1L*, there was overall attenuation of the significance for MI signatures, and associations for 9 genes remained significant at FDR < 0.1 (Additional file 1: Table S6). *APOD* remained significantly downregulated in both early MI (FDR = 0.003, beta/log2FC = − 0.23) and high CAC (FDR = 0.01, beta/log2FC = − 0.21). We hypothesize that *APOD* may represent a novel target for the treatment and prevention of atherosclerotic disease.

Finally, for the 68 coding genes differentially expressed between MI and control at FDR < 0.1, our secondary analysis found four genes also differentially expressed between MI and high CAC at a Bonferroni corrected *p*-value ≤ 0.0007 (0.05/68). Their expression level was changed in the same direction across the three groups. Three genes (*IRF7, HBG2, and HBG1*) were up-regulated in MI compared to high CAC and controls, and one gene (*PEX26*) was down-regulated in MI compared to high CAC and controls.

### Pathway enrichment analysis to identify biological function pathway signatures for early MI

To explore biological functions and pathways in which the gene signatures of MI might act, we found that annotations of the 68 MI genes were highly enriched for a few GO categories including protein complex binding and phosphoprotein (FDR < 0.05), compare to all human genes as background.

Gene set enrichment analysis (GSEA) [28] was conducted to explore for enrichment of gene sets for the up-regulated and down-regulated genes in participants with MI compared to controls. In a total of 3283 gene sets in GSEA C2 category [28] at FDR < 25% enrichment level, 215 gene sets were significantly up-regulated (out of 1938) and eight gene sets were significantly down-regulated (out of 1345). As shown in Table 4 for gene sets significantly enriched at FDR < 5%, all 36 gene sets were up-regulated in MI with a positive Normalized Enrichment Score, including several interferon response pathways, insulin receptor recycling, known targets of transcription factor STAT3, and a gene set related to epigenetic regulation in which 17 genes were significantly silenced by methylation (*p*-value = 0, and FDR q-value = 0.02). The up-regulation of gene expression in the group with early MI compared to controls (Additional file 1: Fig. S4) supports the hypothesis for an epigenetic role in MI pathogenesis.

**Table 4:** Gene set enrichment analysis (GSEA) for enrichment of gene sets/pathways for the up-regulated and down-regulated genes in participants with MI compared to controls

NAME in the Molecular Signatures Database (MSigDB)	SIZE	NES	NOM *p*-val	FDR q-val
BOWIE_RESPONSE_TO_EXTRACELLULAR_MATRIX	17	0.834137	0	0
DAZARD_UV_RESPONSE_CLUSTER_G4	15	0.810541	0	0
BOWIE_RESPONSE_TO_TAMOXIFEN	18	0.800464	0	0
ZHANG_INTERFERON_RESPONSE	22	0.760159	0	5.99E-04
BENNETT_SYSTEMIC_LUPUS_ERYTHEMATOSUS	30	0.730185	0	0.001917
ZHANG_ANTIVIRAL_RESPONSE_TO_RIBAVIRIN_UP	20	0.724651	0	0.002397
CREIGHTON_AKT1_SIGNALING_VIA_MTOR_DN	20	0.687019	0	0.009875
UROSEVIC_RESPONSE_TO_IMIQUIMOD	20	0.687635	0	0.011115
STAMBOLSKY_TARGETS_OF_MUTATED_TP53_DN	38	0.67526	0	0.012077
CHIBA_RESPONSE_TO_TSA_UP	29	0.677011	0	0.012327
DAZARD_UV_RESPONSE_CLUSTER_G24	15	0.680731	0.001965	0.012367
MOSERLE_IFNA_RESPONSE	29	0.670738	0	0.013561
EINAV_INTERFERON_SIGNATURE_IN_CANCER	26	0.662667	0	0.016484
GALE_APL_WITH_FLT3_MUTATED_DN	16	0.652891	0	0.019301
LIANG_SILENCED_BY_METHYLATION_2	32	0.654796	0	0.02017
LIANG_HEMATOPOIESIS_STEM_CELL_NUMBER_QTL	15	0.653422	0.006276	0.020191
JOSEPH_RESPONSE_TO_SODIUM_BUTYRATE_UP	26	0.645603	0	0.021225
REACTOME_TRANSFERRIN_ENDOCYTOSIS_AND_RECYCLING	19	0.646882	0	0.021409
RASHI_NFKB1_TARGETS	18	0.648308	0	0.021688
CAVARD_LIVER_CANCER_MALIGNANT_VS_BENIGN	16	0.639799	0.001927	0.024119
DAUER_STAT3_TARGETS_DN	46	0.63281	0	0.026719
HARRIS_BRAIN_CANCER_PROGENITORS	15	0.633145	0.00404	0.027771
KRASNOSELSKAYA_ILF3_TARGETS_UP	28	0.633243	0	0.029035
RADAEVA_RESPONSE_TO_IFNA1_UP	47	0.626684	0	0.030557
KIM_LRRC3B_TARGETS	28	0.626813	0	0.03178
SUH_COEXPRESSED_WITH_ID1_AND_ID2_UP	16	0.623882	0.005906	0.032143
NOJIMA_SFRP2_TARGETS_DN	18	0.621049	0.002024	0.034003
XU_HGF_TARGETS_INDUCED_BY_AKT1_6HR	16	0.60961	0.008065	0.039922
BROWNE_INTERFERON_RESPONSIVE_GENES	63	0.610055	0	0.040405
REACTOME_INTERFERON_ALPHA_BETA_SIGNALING	46	0.610758	0	0.040506
GRATIAS_RETINOBLASTOMA_16Q24	15	0.611119	0.00813	0.041194
KANG_CISPLATIN_RESISTANCE_UP	16	0.611884	0	0.041448
WELCH_GATA1_TARGETS	18	0.613601	0	0.042096
OUYANG_PROSTATE_CANCER_PROGRESSION_DN	16	0.612352	0.004008	0.042214
REACTOME_INSULIN_RECEPTOR_RECYCLING	17	0.601722	0	0.049199

A total of 3283 gene sets in GSEA C2 category [28] were tested. At FDR < 25% enrichment level, 215 gene sets were significantly up-regulated (out of 1938) and eight gene sets were significantly down-regulated (out of 1345)

*NES* Normalized enrichment score, *NOM p-val* Nominal *p*-value, *FDR q-val* False discovery rate q-value

Other interesting gene sets at significantly enriched FDR < 25% but FDR > 5% include sets for hypoxia, oxidative phosphorylation, inflammatory response, obesity and cholesterol biosynthesis (Additional file 1: Table S7), consistent with a number of pathophysiological pathways previously implicated in coronary artery disease and MI. New knowledge regarding these regulatory changes may improve our ability to functionally characterize susceptibility variants associated with diseases and related risk factors.

### Validation of MI expression signatures using Affymetrix exon-array expression data

Of 198 RNA-Seq samples, there was an overlap of 193 with the previous 5626 Affymetrix Exon-array data. Using these 193 common samples, only one gene (CLDN8, beta = 0.16, *p* = 2.84e-06, FDR = 0.05) was differentially expressed between MI and controls, a finding that was validated in exon array data at FDR < 0.1. For 10,595 expressed genes found in both platforms, we first computed the statistic t value for each gene on each platform by comparing MI cases with controls. We found a significant correlation of the t values between RNA-Seq and exon array (*r* = 0.21, *P* < 1e-324, Additional file 1: Fig. S5), which is consistent with previous reports [30].

**Fig. 3:**
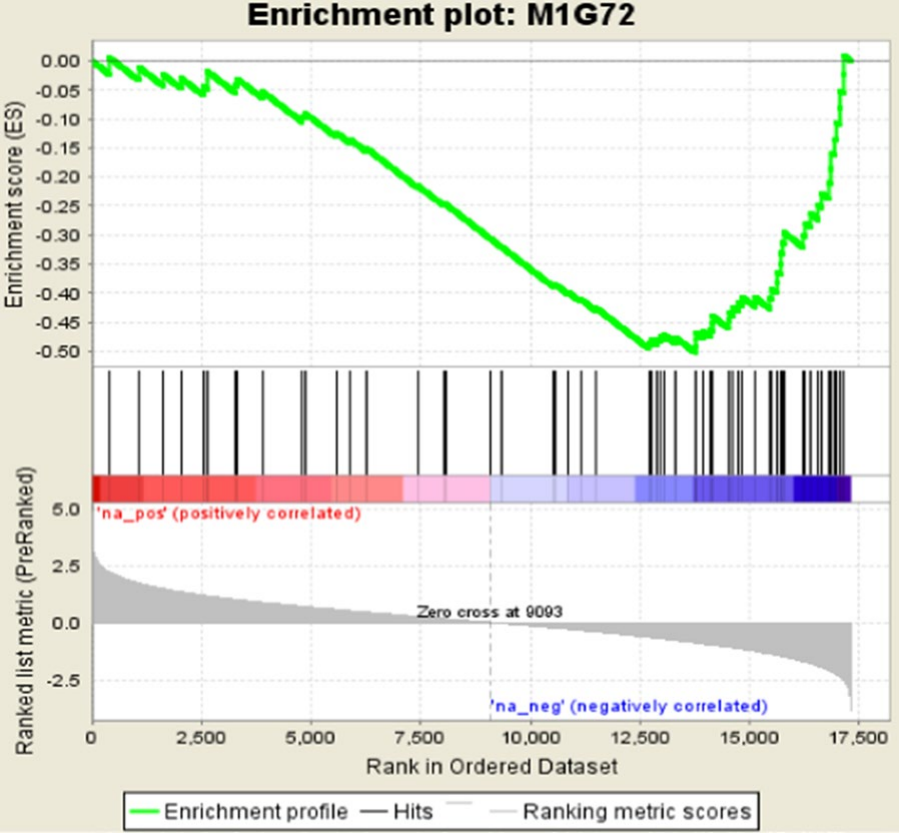
GSEA result of 70 MI genes (21 upregulated, and 49 down-regulated in Table 2), of which 66 unique genes found in Exon array platform. The list of 17,873 coding genes ranked by t value of comparing early MI with control on Affymetrix Exon array data (*N* = 193 samples). Normalized Enrichment Score (NES) = −2.14, Nominal *p*-value = 0 and FDR q-value =0

The replication rate of expression signatures may be low when performing single gene comparisons between different platforms (e.g., RNA-Seq and exon array) [28] or across different high-throughput studies. Therefore, we conducted a GSEA analysis to validate whether the 68 DEG considered as an entire gene set is significantly enriched using exon array MI cases versus controls. For the protein coding genes, 66 unique genes were found on the exon-array. 17,873 exon array genes were ranked by t value (from positive to negative) in the MI case:control comparison. The enrichment is significant (nominal *p*-value < 0.001) with Normalized Enrichment Score = − 2.14 (Fig. 3), indicating overall down-regulation in MI compared with the controls in exon array data, consistent with our findings from RNA-Seq (47 of 68 down-regulated genes). Additional file 1: Table S8 reports 35 leading edge genes among the set of 68 genes. The leading-edge subset can be interpreted as the core group of genes that accounts for the gene set’s enrichment signal [28].

### Independent replication of MI expression signatures using Illumina RNA-Seq in the Rotterdam study cohort

We further replicated our MI gene signatures in an independent cohort, the Rotterdam Cohort study (*N* = 807) in which Illumina RNA-Seq was conducted. Among our 70 MI genes (68 coding and 2 lincRNAs), 60 coding genes were detectable (CPM > 1 in > 10% of samples) and therefore analyzed in the Rotterdam RNA-Seq dataset. We found a significant correlation of the effect size of MI between our discovery FHS RNA-Seq and Rotterdam replication RNA-Seq (Spearman.*r* = 0.53, *P* < 1.3e− 05). Specifically, among 60 genes tested, we found 9 genes were differentially expressed between MI cases (*n* = 28) and controls (*n* = 376) at *P* < 0.05 (Table 2), and 8 were expressed differently in the same direction as indicated in bold in Table 2. In addition, among 8 coding genes supported by *p* value and direction, two genes were also differentially expressed between CHD cases and controls in the same direction (*HNRNPR* with *P* = 0.01, and *NPDC1* with *P* = 0.0026). Furthermore, among 60 genes tested, 77% (46 genes) of the associations are in the same effect direction between MI and controls, indicating consistency of effect, although a larger sample size is needed to replicate significant associations with MI.

## Discussion

Although GWAS have identified many genetic variants associated with MI and subclinical coronary atherosclerosis (e.g. high CAC), the totality of evidence suggests that many GWAS variants are located in non-coding genomic regions. Genes reported for these variants are based on their proximity to nearby genes and limited to annotated protein-coding genes that may not represent the causal genes responsible for the traits studied, and much of the functional genomics of coronary artery disease remains unknown. Whole transcriptome studies using RNA-Seq can simultaneously comprehensively profile both coding and non-coding genes and transcripts associated with disease status, providing new knowledge and functional biological insights into human diseases.

We first systematically characterized expression patterns of coding mRNAs and non-coding lincRNAs in whole blood through a high-coverage of RNA-sequencing experiment (one sample per lane). Much more highly-expressed coding genes are identified comparing to lincRNAs in whole blood, which is consistent with a previous report [32]. When compared to 16 other human tissues, a larger percentage of lincRNAs versus coding genes are expressed especially in whole blood, indicating that lincRNAs might be more tissue/cell-type specific compared to coding genes. Further studies with a greater diversity of tissue/cell data generated from the same research participants are needed to confirm this finding.

We identified coding and non-coding gene expression signatures associated with prior early MI, and a few expression signatures were also discovered for high CAC, a noninvasive measure of coronary atherosclerosis, which precedes most cases of MI [3, 4]. Of note, *APOD*, encoding a component of high density lipoprotein, was expressed significantly lower in both early MI (FDR = 0.007) and in high CAC (FDR = 0.01) compared with controls, respectively. Altered expression of *APOD* was not reported to be significantly associated with coronary heart disease in our prior FHS investigation [35], but the cases were not of early onset and only half of the cases had prior MI. Furthermore, a prior separate FHS investigation found that protein level of APOD is also decreased in MI new-onset patients compared to controls [36], providing orthogonal evidence for APOD as an attractive novel candidate for clinical and subclinical atherosclerosis. Tsukamoto et al. reported altered response to myocardial infarction in Apod knockout mice [37], revealing *APOD* as a cardioprotective gene using a mouse model of lethal atherosclerotic coronary artery disease. In addition, high levels of APOD protein in humans are associated with protective inflammatory levels and fatty liver in initial human studies [38, 39]. Further investigation of APOD in mouse models and larger human studies will be needed for experimental validation of this mechanism, and to allow investigation of underlying mechanisms related to atherosclerotic coronary artery disease.

In addition, despite our relatively modest sample size, we identified 71 gene expression signatures for MI and CAC in whole blood. Pathway analysis for these genes highlighted immune response, lipid metabolic processes, and interferon regulatory factor as potential pathways involved in disease progress/pathogenesis, consistent with the known pathophysiology of coronary artery disease.

Only a few lincRNA expression signatures were found to be associated in either MI or CAC, which might be due to a relatively small sample size and much lower abundance of lincRNAs in human tissues. Because we undertook deep sequencing coverage, we were able to identify many lincRNA specifically expressed in blood that are likely not reliably detected in lower coverage RNA-Seq experiments. The lincRNA RP11-245 J9.5 associated with CAC is of interest. It expressed high in peripheral blood and log2FC = − 0.6 (Fig. 2d). This gene is also known as PSMD6-AS2, a gene that could disrupt expression of a proteasome subunit. This gene was identified as differentially expressed in a study of atherosclerotic macrophages [40]. Another proteasomal subunit, PSMC3 mutation is associated with subcutaneous calcifications [41], indicating that this lincRNA RP11-245 J9.5 might be involved in atherosclerosis by regulating several proteasome subunits. However, further replication of its association with CAC in independent studies is needed to warrant future experiment mechanism studies of this lincRNA and identification of its functional targets. LincRNA may act as key transcriptional regulators in different stages of biological systems, from chromatin regulation to transcription regulation [22]. Future studies are warranted to explore the relationship between these lincRNA signatures and their regulated mRNA targets and specific biological process in atherosclerosis, and the implications for new therapeutic targets for treatment and prevention of clinical MI and subclinical atherosclerosis.

Pathway enrichment analysis identified interesting results including a pathway/gene set called “STAT3_TARGETS_DN”. Signal transducer and activator of transcription 3 (STAT3) protein has been linked to cardiovascular disease through multiple pathways in experimental and animal studies [42, 43]. STAT3 is a key regulator of cell-to-cell communication in the heart, modulates proliferation, differentiation, survival, oxidative stress, and/or metabolism in cardiomyocytes, fibroblasts, endothelial cells, progenitor cells, and various inflammatory cells [44]. It has been well documented that monocytes and macrophages produce inflammatory cytokines to repair the injury during myocardial infarction and hypertrophy [45]. The early activation of STAT3 during diseased stage could be the protective response of system to reduce the cardiac death and remodeling through transcription factor STAT3 binds to promoter region of cardio-protective genes in nucleus [43].

In our study, we have adjusted for well-known risk factors for MI and subclinical atherosclerosis. Future studies in larger cohorts with clinically apparent coronary heart disease or subclinical atherosclerosis will allow exploration of the role of specific risk factors in the progression of subclinical atherosclerosis to clinical atherosclerosis at the molecular level. In addition to our modest sample size, the major limitation of our study is the use of whole blood RNA, which includes heterogeneity of leukocyte cell types, although we adjusted for differences of cell types in our study. Future RNA-Seq experiments in affected tissues/cells such as atherosclerotic aortic root cells and coronary artery endothelial cells are warranted.

While we acknowledge there are limitations to our pilot study, we believe we have identified several lessons for future applications of whole blood RNA sequencing for discovery of coronary atherosclerosis genes. Among the limitations, RNA-Seq experiments are still costly, and as with our study, the resulting relatively small sample size of these experiments may continue to limit statistical power to study rare RNA species such as lincRNAs that require deep coverage sequencing. Further, non-strand-specific RNA-Seq protocol limits accurate discovery of antisense transcripts and might lead to bias for quantifying genes that overlap with anti-sense transcripts. Finally, unmeasured confounders may affect results of association studies. Nevertheless, we conclude that blood RNA sequencing analysis is feasible and may detect a much fuller and informative spectrum of gene expression than is seen on gene chip arrays, although careful RNA extraction and high resolution sequencing will be required for early studies.

## Conclusion

In summary, we identified significant MI-specific expression signatures, with eight genes (15%) supported in an independent cohort with RNA-Seq data. Of note, *APOD*, encoding a component of high-density lipoprotein, was significantly downregulated in both early MI and in high CAC compared with controls, indicating a novel candidate target for the treatment and prevention of atherosclerotic disease. Our findings provide insights into mechanisms through which transcriptome-level variation may influence the development of subclinical coronary atherosclerosis and, ultimately, clinical MI.

## Supplementary Information

**Additional file 1.** Supplementary Material including Methods, Figures and Tables.

## Data Availability

The expression levels for these 25,593 coding genes and 8,267 lincRNAs were deposited in the NCBI database of genotypes and phenotypes (dbGaP) under the Dataset Accession ID (phs000007).

## Abbreviations

CAC: Coronary artery calcification
CPM: Counts per million mapped reads
DEGs: Differentially expressed genes
FDR: False discovery rate
FHS: Framingham Heart Study
FPKM: Fragments per kilobase of transcript per million mapped reads
GSEA: Gene set enrichment analysis
GWAS: Genome-wide association studies
lincRNAs: Long intergenic noncoding RNAs
MI: Myocardial infarction
PCA: Principle component analysis
RIN: RNA integrity number
