# Supplementary material for: Genome-wide transcriptome study using deep RNA sequencing for myocardial infarction and coronary artery calcification
Source: BMC Med Genomics. 2021 Feb 10;14:45. doi: 10.1186/s12920-020-00838-2 (PMC7874462; doi:10.1186/s12920-020-00838-2)
Supplement: Supplementary file 1 — Additional file 1. Supplementary Material including Methods, Figures and Tables. [file 12920_2020_838_MOESM1_ESM.pdf]

SUPPLEMENTARY MATERIAL

Supplementary Figures

Supplementary Figure 1. RNA-Sequencing reads mapping statistics

Supplementary Figure 2a. A heatmap of expression profiles of 52 coding genes between blood and Human BodyMap tissues (n=16).

Supplementary Figure 2b. A heatmap of expression profiles of 36 lincRNAs between blood and Human BodyMap tissues (n=16).

Supplementary Figure 3a. Correlation plot between RNA-Seq and Affymetrix Exon-array platforms for one sample.

Supplementary Figure 3b. The histogram distribution of r value for all 193 samples.

Supplementary Figure 4. GSEA results for the gene set named "LIANG_SILENCED_BY_METHYLATION_2" (Nominal p-value = 0, and FDR q-value = 0.02) in which 17 genes silenced by methylation.

Supplementary Figure 5. The Pearson correlation of t values comparing cases of MI (i.e., Case 1) with controls for 10,595 expressed genes found in both platforms

Supplementary Tables

Supplementary Table 1. RNA quality of the studied samples across three groups

Supplementary Table 2. Nine samples sequenced twice

Supplementary Table 3. Protein-coding genes were highly expressed in whole blood (435 genes with log2 (FPKM) > 4 in all samples)

Supplementary Table 4. GO analysis of 435 protein-coding genes

Supplementary Table 5. LincRNAs highly expressed in whole blood (36 lincRNAs with log2 (FPKM) > 1 in all samples)

Supplementary Table 6. Results of adjusting for additional 9 clinical covariates for 68 coding gene and 2 lincRNA signatures identified by Model 1.

Supplementary Table 7. GSEA pathway analysis and leading-edge genes (FDR<0.25 including 215 gene sets are upregulated in phenotype MI, and 8 gene sets are upregulated in controls)

Supplementary Table 8. 35 leading edge genes among the gene set of 72 genes on Affymetrix Exon-array data

Supplementary Methods

Rotterdman RNA-Seq cohort includes sample size, RNA library preparation, sequencing, and data processing and data analysis

Rotterdam Study RNA-Seq data was generated as part of the BIOS project and is described in detail elsewhere[1]. In short, Total RNA was globin cleared using Ambion GLOBINclear and sequenced to a minimum yield of 15M paired-end reads on an HiSeq 2000. Data was aligned to reference genome hg19 using STAR, followed by quantification of all GENCODE genes using custom scripts. We then extracted the counts of 56,515 transcripts for 807 participants of the Rotterdam Study (RS-I, II and III). Using edgeR, CPM values were generated for each sample, and transcripts with CPM<1 in more than 90% of samples were excluded, allowing 15,331 transcripts in further analysis.

For each coded phenotype, linear regression analysis was performed in R, correcting for age, gender, flowcell and the number of sequenced reads. A custom linear regression

script was used as the dataset was too large to be processed by edgeR or DEseq. The effect sizes and uncorrected p-values were reported for each of the candidate genes of the discovery analysis.

Rotterdam Study CAC data

The Rotterdam Study is a population-based cohort study that started in 1990 (PMID: 26386597). All inhabitants aged 55 years and older, living in a suburb of Rotterdam were invited and 7983 agreed to participate (78%). This group is referred to as Rotterdam Study cohort I (RS-I). In 2000, a second study cohort was introduced (RS-II) comprising 3011 persons.

CAC was measured in the epicardial coronary arteries using an EBCT with a C-150 Imatron scanner (GE-Imatron, San Francisco, California) in the third visit of the original cohort (RS-I) and the first visit of the extended cohort (RS-II) (PMID: 20946998). Participants exercised adequate breath-holding before the scan. Thirty-eight images were obtained from the level of the root of the aorta through the heart with 100-ms scan time and 3-mm slice thickness. Images were acquired at 80% of the cardiac cycle, using electrocardiogram triggering, during a single breath-hold. Quantification of CAC was performed with AccuImage software (AccuImage Diagnostics Corporation), displaying all pixels with a density above 130 Hounsfield Units (HU). A calcification was defined as a minimum of 2 adjacent pixels with a density over 130 HU[2].

CAC was assessed by a 16-slice or 64-slice MDCT scanners (SOMATOM Sensation 16 or 64, Siemens, Forcheim, Germany) in the fourth visit of the original cohort (RS-I) and the second visit of the extended cohort (RS-II)[3]. The cardiac scan

reached from the apex of the heart to the tracheal bifurcation. Atherosclerotic calcification was identified based on a threshold of 130 HU, using dedicated software (Syngo Calcium Scoring, Siemens, Forcheim, Germany). Calcification was quantified with the Agatston score using dedicated software (syngo Calcium Scoring, Siemens, Forcheim, Germany). The total score per vascular bed was calculated by adding the scores of all lesions in that bed[3].

References

1. Zhernakova, D.V. *et al.* Identification of context-dependent expression quantitative trait loci in whole blood. *Nat Genet* **49**, 139-145 (2017).
2. Elias-Smale, S.E. *et al.* Coronary calcium score improves classification of coronary heart disease risk in the elderly: the Rotterdam study. *J Am Coll Cardiol* **56**, 1407-14 (2010).
3. Elias-Smale, S.E. *et al.* Burden of atherosclerosis improves the prediction of coronary heart disease but not cerebrovascular events: the Rotterdam Study. *Eur Heart J* **32**, 2050-8 (2011).

Supplemental Figure 1. RNA-Sequencing reads mapping statistics

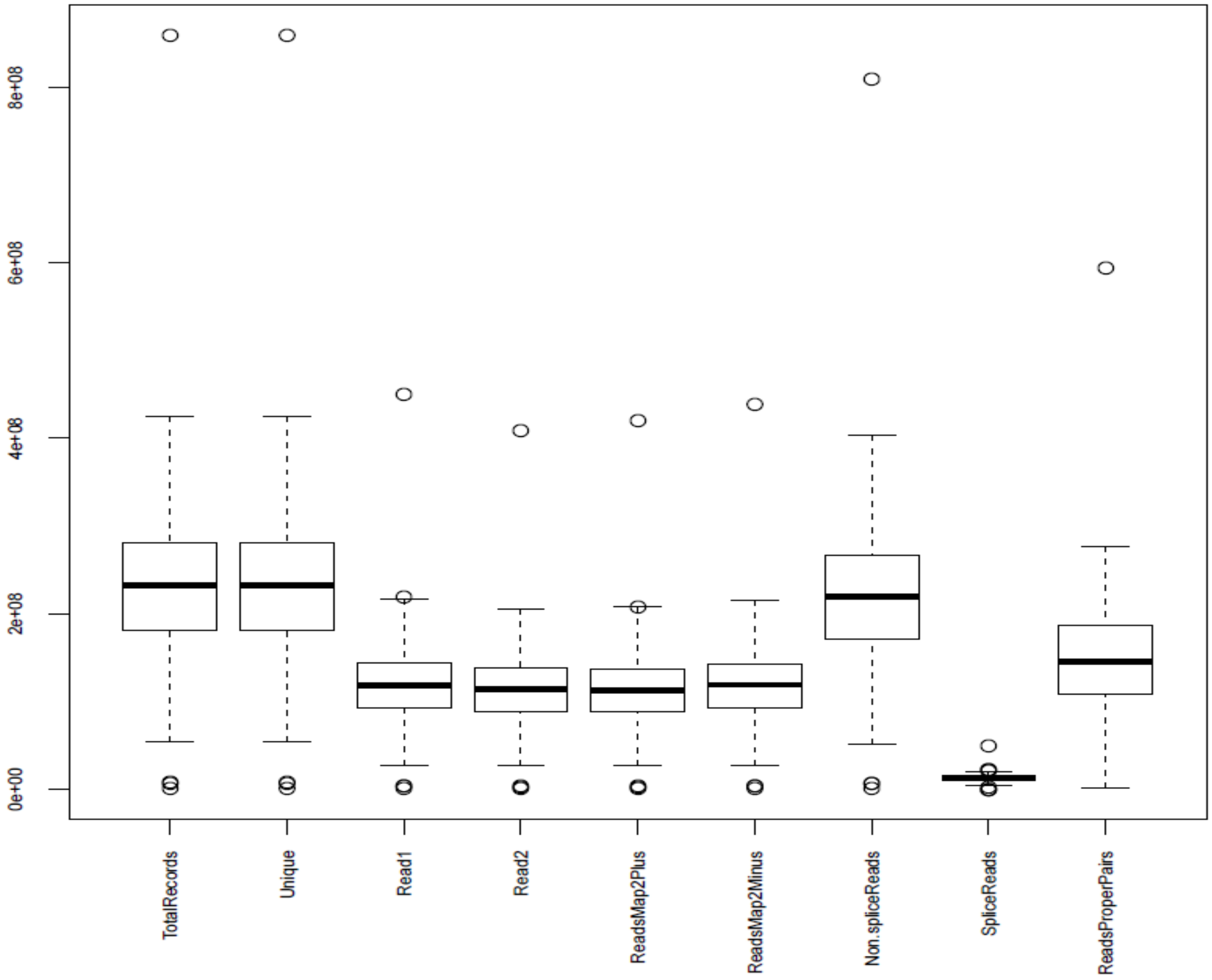

Supplemental Figure 2a. A heatmap of expression profiles of 52 coding genes between blood and Human BodyMap tissues (n=16). Half of the coding genes are expressed higher in blood than in 16 other tissues in BodyMap RNA-seq data (top and middle panels).

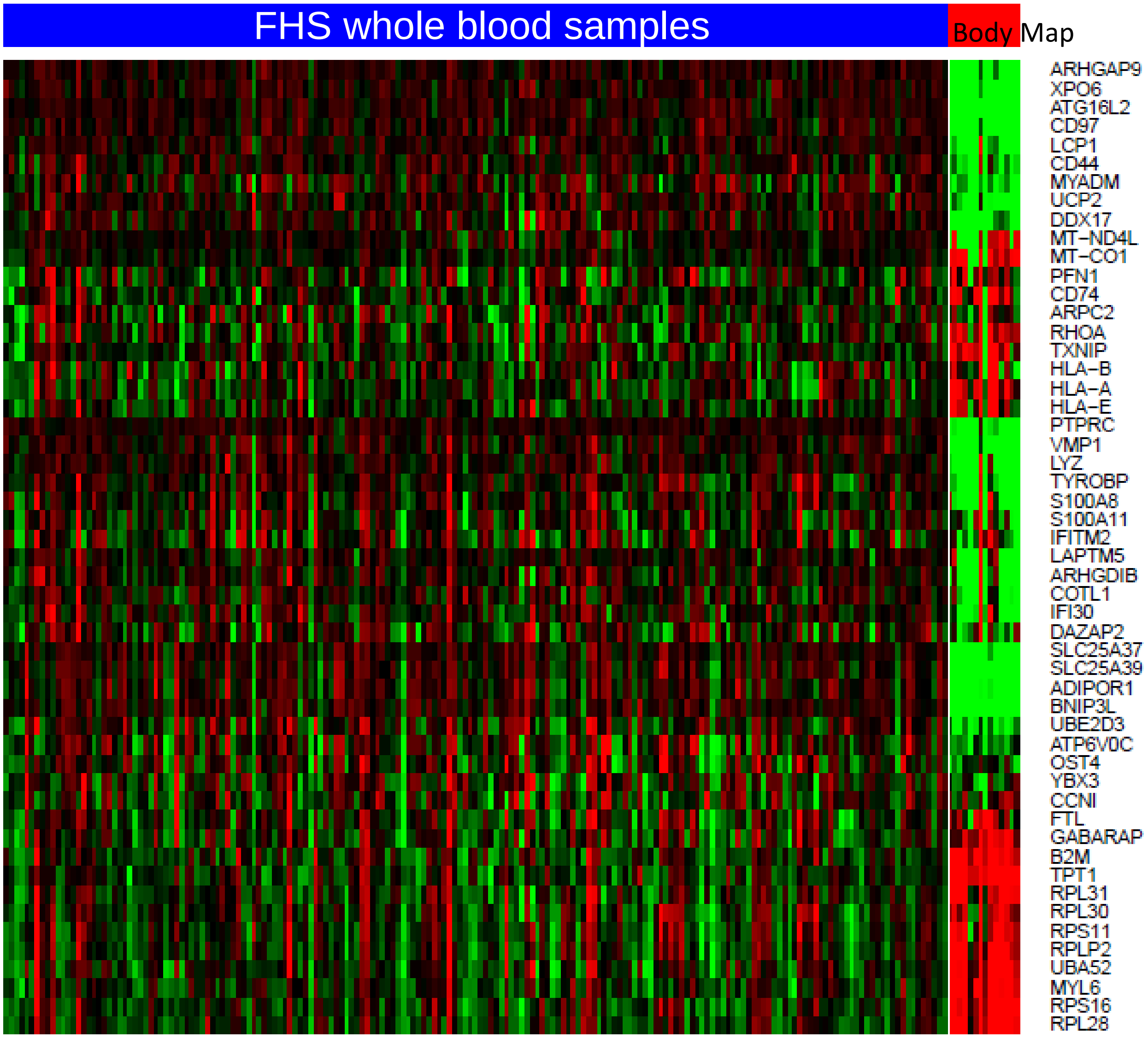

Supplemental Figure 2b. A heatmap of expression profiles of 36 lincRNAs between blood and Human BodyMap tissues (n=16). The majority of lincRNAs (26/36=72%) were expressed higher in blood than in 16 other tissues in the BodyMap RNA-seq data (top panel).

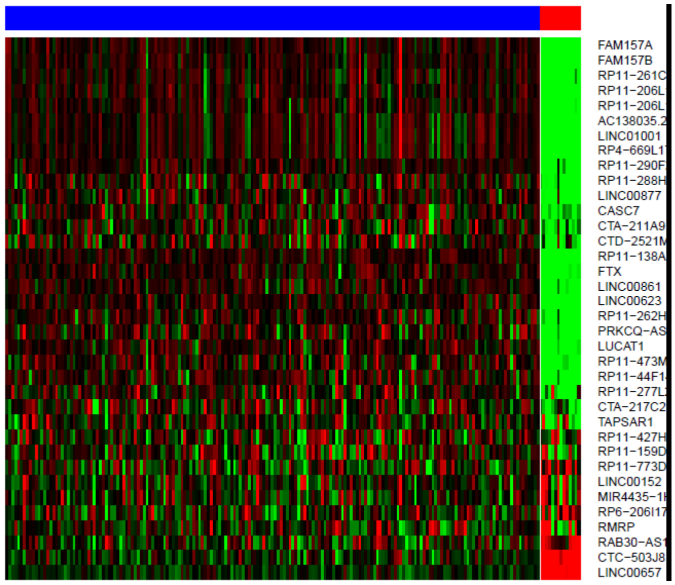

Supplemental Figure 3a. Correlation plot between RNA-Seq and Affymetrix Exon-array platforms for one sample.

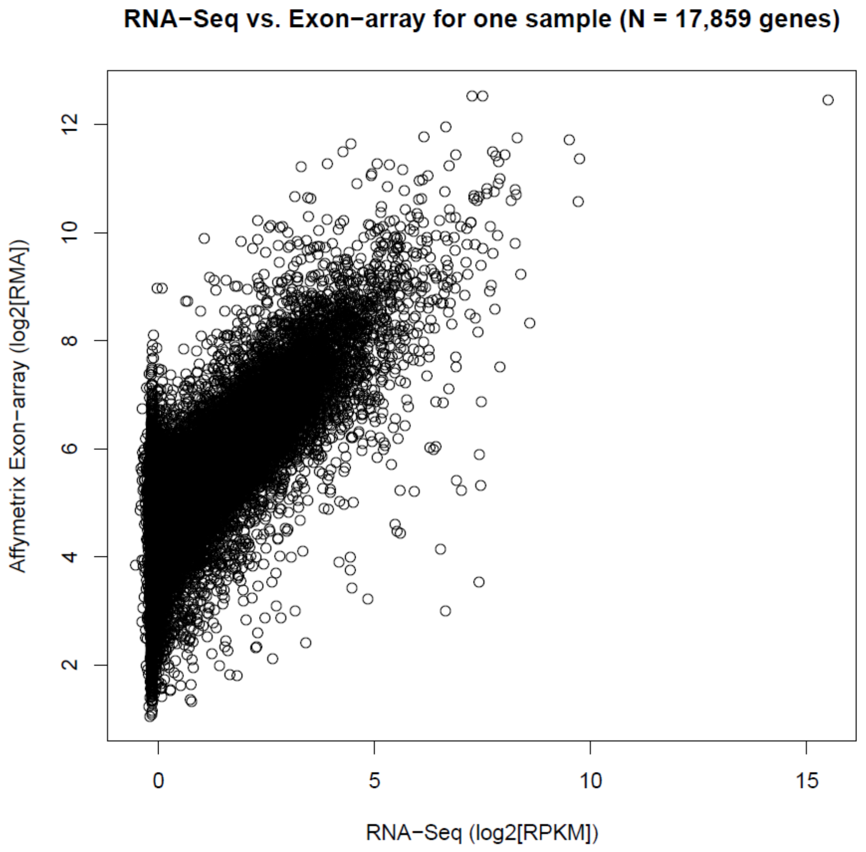

Supplemental Figure 3b. The histogram distribution of r value for all 193 samples.

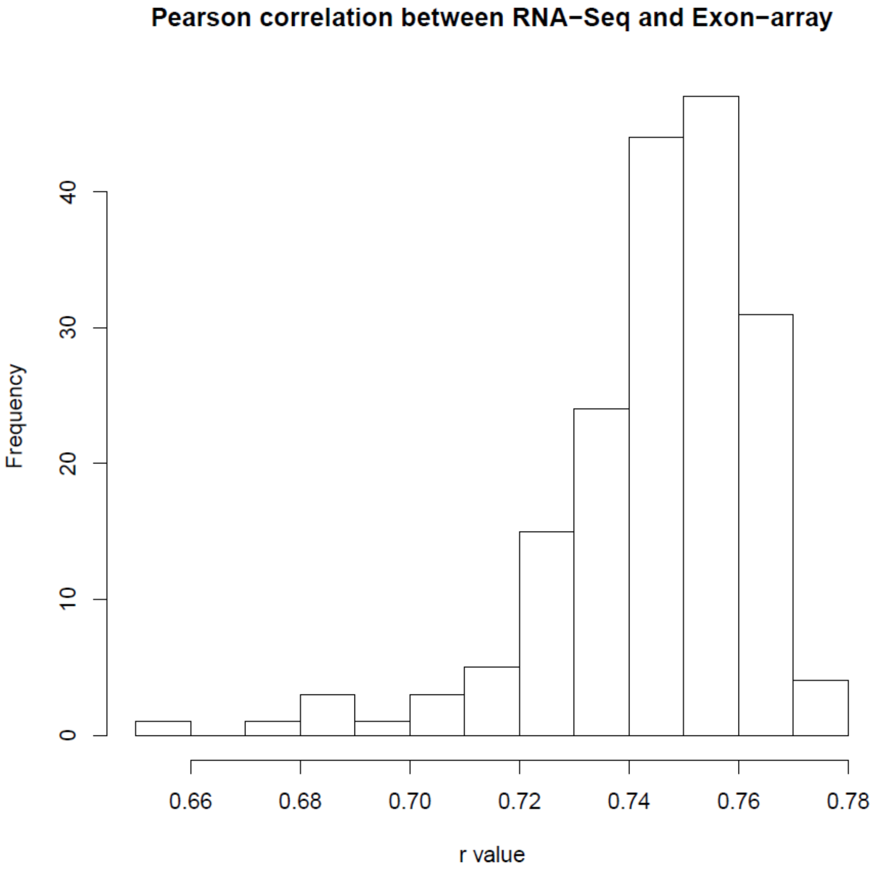

Supplemental Figure 4. GSEA results for the gene set named "LIANG_SILENCED_BY_METHYLATION_2" (Nominal p-value = 0, and FDR q-value = 0.02) in which 17 genes silenced by methylation.

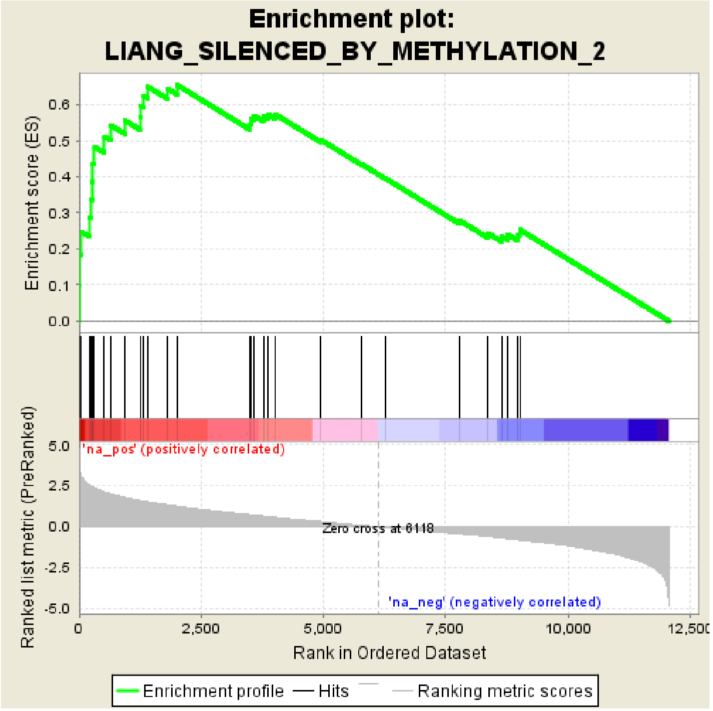

	PROBE	GENE SYMBOL	GENE_TITLE	RANK IN GENE LIST	RANK METRIC SCORE	RUNNING ES	CORE ENRICHMENT
1	IRF7	IRF7 Entrez, Source	interferon regulatory factor 7	1	4.708	0.0990	Yes
2	IFI6	IFI6 Entrez, Source	interferon, alpha-inducible protein 6	3	4.132	0.1859	Yes
3	IFIT1	IFIT1 Entrez, Source	interferon-induced protein with tetratricopeptide repeats 1	55	3.082	0.2466	Yes
4	SLPI	SLPI Entrez, Source	secretory leukocyte peptidase inhibitor	209	2.542	0.2874	Yes
5	IFI44	IFI44 Entrez, Source	interferon-induced protein 44	239	2.489	0.3374	Yes
6	S100A4	S100A4 Entrez, Source	S100 calcium binding protein A4	270	2.444	0.3863	Yes
7	MX1	MX1 Entrez, Source	myxovirus (influenza virus) resistance 1, interferon-inducible protein p78 (mouse)	283	2.415	0.4362	Yes
8	IFI44L	IFI44L Entrez, Source	interferon-induced protein 44-like	316	2.368	0.4834	Yes
9	MX2	MX2 Entrez, Source	myxovirus (influenza virus) resistance 2 (mouse)	517	2.125	0.5115	Yes
10	PI3	PI3 Entrez, Source	peptidase inhibitor 3, skin-derived (SKALP)	651	2.002	0.5425	Yes
11	XAF1			946	1.780	0.5556	Yes
12	OAS1	OAS1 Entrez, Source	2',5'-oligoadenylate synthetase 1, 40/46kDa	1247	1.600	0.5643	Yes
13	ICAM1	ICAM1 Entrez, Source	intercellular adhesion molecule 1 (CD54), human rhinovirus receptor	1261	1.591	0.5967	Yes
14	TNFAIP3	TNFAIP3 Entrez, Source	tumor necrosis factor, alpha-induced protein 3	1325	1.563	0.6244	Yes
15	IFIT3	IFIT3 Entrez, Source	interferon-induced protein with tetratricopeptide repeats 3	1408	1.522	0.6496	Yes
16	OAS2	OAS2 Entrez, Source	2'-5'-oligoadenylate synthetase 2, 69/71kDa	1805	1.337	0.6448	Yes
17	THBD	THBD Entrez, Source	thrombomodulin	2004	1.255	0.6548	Yes

Supplemental Figure 5. The Pearson correlation of t values comparing cases of MI (i.e., Case 1) with controls for 10,595 expressed genes found in both platforms; the overall correlation between RNA-Seq and exon array is modest but significant (r = 0.21, P<1e-324).

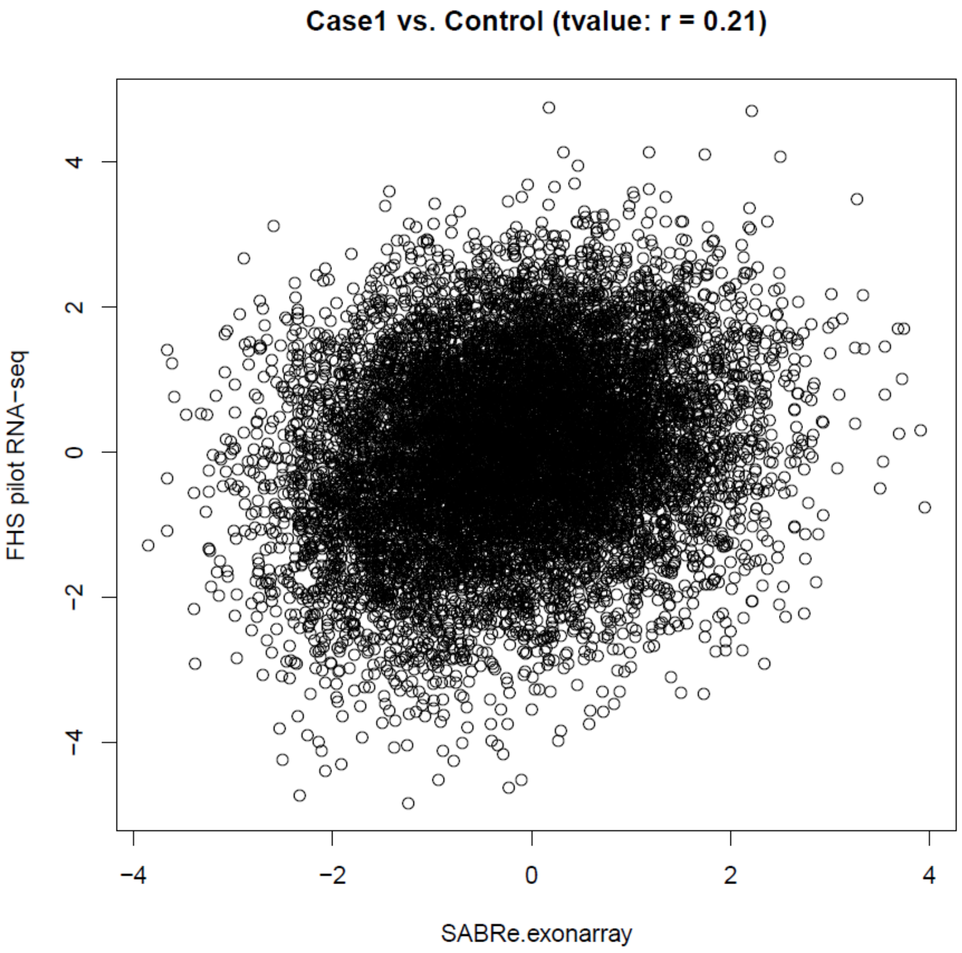

Supplemental Table 1. RNA quality of the studied samples across three groups.

N=198	Early MI (n=55)	High CAC w/o MI (n=72)	Controls (n=71)	P-value
**RNAQualityScore (RIN)**	6.84 ± 2.02	6.74 ± 1.94	7 ± 1.41	0.68
**Concentration (ng/ul)**	105.30 ± 81.08	134.1 ± 64.83	142.20 ± 92.15	0.03
**Yield (ug)**	4.21 ± 3.24	5.37 ± 2.59	5.69 ± 3.69	0.03
**X260_280**	1.54 ± 1.19	1.72 ± 0.27	1.73 ± 0.30	0.23

Supplemental Table 2. Samples sequenced twice (N=9)

						Correlation between samples sequenced twice	
**Sample_Id**	**Reads**	**Mapped**	**Mapping rate**	**Aligned pairs**	**Concordant pair alignment rate**	**logCPM**	**logFPKM**
FHSB13_8	147,401,552	50,019,054	0.329	40,719,199	0.25	**0.77**	0.71
FHSB4_3	186,590,516	103,101,639	0.537	74,735,840	0.361	**0.91**	0.83
FHSB2_12	110,841,030	72,439,633	0.65	55,436,065	0.441	**0.99**	0.91
FHSB2_13	100,667,196	71,720,890	0.708	56,555,404	0.49	**0.95**	0.84
FHSB2_10	83,276,638	62,516,446	0.746	50,352,216	0.535	**0.98**	0.87
FHSB10_15	71,363,082	60,014,886	0.831	51,534,020	0.654	**0.93**	0.83
FHSB1_4	166,226,536	850,905	0.005	601,967	0.003	**0.81**	0.75
FHSB4_4	200,911,973	3,484,644	0.017	2,788,914	0.013	**0.78**	0.65
FHSB4_9	137,614,821	3,970,270	0.028	3,225,507	0.021	**0.91**	0.77

Supplemental Table 3. Protein-coding genes were highly expressed in whole blood (435 genes with log2 (FPKM) > 4 in all samples)

gene_id	gene_short_name	locus	gene_biotype
ENSG00000000938	FGR	1:27938574-27961788	protein_coding
ENSG00000002834	LASP1	17:37026111-37078023	protein_coding
ENSG00000003402	CFLAR	2:201980826-202041410	protein_coding
ENSG00000003756	RBM5	3:50126340-50156454	protein_coding
ENSG00000005483	KMT2E	7:104654625-104754808	protein_coding
ENSG00000005844	ITGAL	16:30483978-30534506	protein_coding
ENSG00000008988	RPS20	8:56979853-56987069	protein_coding
ENSG00000010244	ZNF207	17:30677135-30708905	protein_coding
ENSG00000010404	IDS	X:148558520-148602635	protein_coding
ENSG00000011600	TYROBP	19:36395302-36399197	protein_coding
ENSG00000012779	ALOX5	10:45869660-45941561	protein_coding
ENSG00000013306	SLC25A39	17:42396992-42402238	protein_coding
ENSG00000015285	WAS	X:48534984-48549818	protein_coding
ENSG00000018280	SLC11A1	2:219246751-219261617	protein_coding
ENSG00000019582	CD74	5:149781199-149792492	protein_coding
ENSG00000025708	TYMP	22:50964180-50968485	protein_coding
ENSG00000026025	VIM	10:17270257-17279592	protein_coding
ENSG00000026297	RNASET2	6:167342991-167370679	protein_coding
ENSG00000026508	CD44	11:35160416-35253949	protein_coding
ENSG00000034510	TMSB10	2:85132748-85133795	protein_coding
ENSG00000034713	GABARAPL2	16:75600248-75611779	protein_coding
ENSG00000038427	VCAN	5:82767283-82878122	protein_coding
ENSG00000043462	LCP2	5:169673240-169725231	protein_coding
ENSG00000048740	CELF2	10:11047258-11378674	protein_coding
ENSG00000051523	CYBA	16:88709690-88717560	protein_coding
ENSG00000059728	MXD1	2:70124819-70170077	protein_coding
ENSG00000060138	YBX3	12:10851682-10875911	protein_coding
ENSG00000060237	WNK1	12:861758-1020618	protein_coding
ENSG00000062716	VMP1	17:57784552-57919616	protein_coding

ENSG00000063177	RPL18	19:49118584-49122793	protein_coding
ENSG00000064601	CTSA	20:44518782-44527459	protein_coding
ENSG00000064666	CNN2	19:1026297-1039068	protein_coding
ENSG00000065357	DGKA	12:56321102-56347811	protein_coding
ENSG00000065413	ANKRD44	2:197831740-198175897	protein_coding
ENSG00000066926	FECH	18:55215514-55254004	protein_coding
ENSG00000067182	TNFRSF1A	12:6437922-6451280	protein_coding
ENSG00000067560	RHOA	3:49396577-49450431	protein_coding
ENSG00000070756	PABPC1	8:101698043-101735037	protein_coding
ENSG00000071082	RPL31	2:101618176-101640494	protein_coding
ENSG00000072135	PTPN18	2:131113579-131132982	protein_coding
ENSG00000072778	ACADVL	17:7120443-7128592	protein_coding
ENSG00000072786	STK10	5:171469076-171615390	protein_coding
ENSG00000073921	PICALM	11:85668726-85780924	protein_coding
ENSG00000075415	SLC25A3	12:98987368-98995946	protein_coding
ENSG00000075420	FNDC3B	3:171757417-172119455	protein_coding
ENSG00000075785	RAB7A	3:128444964-128533639	protein_coding
ENSG00000076662	ICAM3	19:10444451-10450499	protein_coding
ENSG00000076770	MBNL3	X:131503344-131623996	protein_coding
ENSG00000076928	ARHGEF1	19:42387227-42434302	protein_coding
ENSG00000076944	STXBP2	19:7701766-7712759	protein_coding
ENSG00000077454	LRCH4	7:100169854-100183776	protein_coding
ENSG00000078304	PPP2R5C	14:102228134-102394326	protein_coding
ENSG00000081237	PTPRC	1:198607800-198726545	protein_coding
ENSG00000083845	RPS5	19:58897766-58906173	protein_coding
ENSG00000084070	SMAP2	1:40810521-40888998	protein_coding
ENSG00000084234	APLP2	11:129939731-130014699	protein_coding
ENSG00000085265	FCN1	9:137801430-137809809	protein_coding
ENSG00000085514	PILRA	7:99965152-99997719	protein_coding
ENSG00000087086	FTL	19:49468557-49470135	protein_coding
ENSG00000087460	GNAS	20:57414772-57486247	protein_coding
ENSG00000090013	BLVRB	19:40953695-40971747	protein_coding

ENSG00000090238	YPEL3	16:30103634-30108236	protein_coding
ENSG00000090382	LYZ	12:69742120-69748014	protein_coding
ENSG00000092010	PSME1	14:24605366-24608176	protein_coding
ENSG00000092199	HNRNPC	14:21677294-21737653	protein_coding
ENSG00000092841	MYL6	12:56551944-56557280	protein_coding
ENSG00000099622	CIRBP	19:1259383-1274879	protein_coding
ENSG00000099875	MKNK2	19:2037469-2051243	protein_coding
ENSG00000100201	DDX17	22:38879444-38903665	protein_coding
ENSG00000100225	FBXO7	22:32870662-32894818	protein_coding
ENSG00000100316	RPL3	22:39708886-39716394	protein_coding
ENSG00000100345	MYH9	22:36677326-36784063	protein_coding
ENSG00000100365	NCF4	22:37257029-37274057	protein_coding
ENSG00000100368	CSF2RB	22:37309669-37336491	protein_coding
ENSG00000100650	SRSF5	14:70193616-70238722	protein_coding
ENSG00000101109	STK4	20:43595114-43708600	protein_coding
ENSG00000101307	SIRPB1	20:1544166-1600707	protein_coding
ENSG00000101596	SMCHD1	18:2655736-2805015	protein_coding
ENSG00000101608	MYL12A	18:3247478-3256234	protein_coding
ENSG00000101782	RIOK3	18:21032786-21066567	protein_coding
ENSG00000102096	PIM2	X:48770458-48776301	protein_coding
ENSG00000102879	CORO1A	16:30194147-30200397	protein_coding
ENSG00000103187	COTL1	16:84599199-84651683	protein_coding
ENSG00000103657	HERC1	15:63900816-64126141	protein_coding
ENSG00000104133	SPG11	15:44854893-44955876	protein_coding
ENSG00000104529	EEF1D	8:144661866-144681711	protein_coding
ENSG00000104765	BNIP3L	8:26240413-26363152	protein_coding
ENSG00000104894	CD37	19:49838427-49846592	protein_coding
ENSG00000105193	RPS16	19:39923846-39926588	protein_coding
ENSG00000105221	AKT2	19:40736223-40791443	protein_coding
ENSG00000105372	RPS19	19:42363987-42376994	protein_coding
ENSG00000105483	CARD8	19:48684026-48759203	protein_coding
ENSG00000106829	TLE4	9:82186687-82341658	protein_coding

ENSG00000106948	AKNA	9:117096435-117156685	protein_coding
ENSG00000108107	RPL28	19:55896712-55914612	protein_coding
ENSG00000108298	RPL19	17:37356535-37360980	protein_coding
ENSG00000108518	PFN1	17:4848946-4852356	protein_coding
ENSG00000108654	DDX5	17:62495733-62504317	protein_coding
ENSG00000108669	CYTH1	17:76670129-76778379	protein_coding
ENSG00000108946	PRKAR1A	17:66507920-66547460	protein_coding
ENSG00000109046	WSB1	17:25621101-25640657	protein_coding
ENSG00000109062	SLC9A3R1	17:72744790-72765492	protein_coding
ENSG00000109332	UBE2D3	4:103715539-103790053	protein_coding
ENSG00000109971	HSPA8	11:122928196-122933938	protein_coding
ENSG00000110321	EIF4G2	11:10818596-10830657	protein_coding
ENSG00000110324	IL10RA	11:117857062-117872196	protein_coding
ENSG00000110367	DDX6	11:118620033-118661858	protein_coding
ENSG00000110700	RPS13	11:17095935-17099334	protein_coding
ENSG00000110713	NUP98	11:3692312-3819022	protein_coding
ENSG00000110719	TCIRG1	11:67806482-67818362	protein_coding
ENSG00000110876	SELPLG	12:109015685-109027735	protein_coding
ENSG00000110955	ATP5B	12:57031958-57039798	protein_coding
ENSG00000111348	ARHGDIB	12:15094950-15114662	protein_coding
ENSG00000111640	GAPDH	12:6643092-6647537	protein_coding
ENSG00000111679	PTPN6	12:7055630-7070479	protein_coding
ENSG00000112096	SOD2	6:160090088-160183561	protein_coding
ENSG00000112303	VNN2	6:133065008-133084598	protein_coding
ENSG00000112306	RPS12	6:133135579-133138703	protein_coding
ENSG00000112576	CCND3	6:41902670-42018095	protein_coding
ENSG00000113732	ATP6V0E1	5:172410759-172462448	protein_coding
ENSG00000113916	BCL6	3:187439164-187463515	protein_coding
ENSG00000114353	GNAI2	3:50263723-50296787	protein_coding
ENSG00000114978	MOB1A	2:74379654-74406025	protein_coding
ENSG00000115091	ACTR3	2:114647536-114720173	protein_coding
ENSG00000115268	RPS15	19:1438357-1440583	protein_coding

ENSG00000115524	SF3B1	2:198254507-198299815	protein_coding
ENSG00000115935	WIPF1	2:175424299-175547644	protein_coding
ENSG00000116701	NCF2	1:183524697-183560011	protein_coding
ENSG00000116741	RGS2	1:192778168-192781403	protein_coding
ENSG00000117289	TXNIP	1:145438468-145442635	protein_coding
ENSG00000117335	CD46	1:207925401-207968858	protein_coding
ENSG00000117410	ATP6V0B	1:44440158-44443967	protein_coding
ENSG00000117616	C1orf63	1:25568727-25664704	protein_coding
ENSG00000118181	RPS25	11:118886421-118889401	protein_coding
ENSG00000118816	CCNI	4:77968310-77997158	protein_coding
ENSG00000119314	PTBP3	9:114980714-115095947	protein_coding
ENSG00000120071	KANSL1	17:44107281-44302733	protein_coding
ENSG00000121060	TRIM25	17:54965269-54991399	protein_coding
ENSG00000121104	FAM117A	17:47787693-47866542	protein_coding
ENSG00000121966	CXCR4	2:136871918-136875735	protein_coding
ENSG00000122406	RPL5	1:93297581-93307481	protein_coding
ENSG00000122566	HNRNPA2B1	7:26229546-26241149	protein_coding
ENSG00000122862	SRGN	10:70847861-70864567	protein_coding
ENSG00000123146	CD97	19:14491312-14519537	protein_coding
ENSG00000123329	ARHGAP9	12:57866037-57882597	protein_coding
ENSG00000123338	NCKAP1L	12:54891494-54937726	protein_coding
ENSG00000123349	PFDN5	12:53689074-53693234	protein_coding
ENSG00000123405	NFE2	12:54685894-54694905	protein_coding
ENSG00000123416	TUBA1B	12:49521564-49525180	protein_coding
ENSG00000124357	NAGK	2:71291473-71306935	protein_coding
ENSG00000124831	LRRFIP1	2:238536218-238722325	protein_coding
ENSG00000125347	IRF1	5:131817300-131826490	protein_coding
ENSG00000125505	MBOAT7	19:54677106-54693733	protein_coding
ENSG00000125818	PSMF1	20:1093905-1160596	protein_coding
ENSG00000125952	MAX	14:65472891-65569413	protein_coding
ENSG00000126247	CAPNS1	19:36630476-36641255	protein_coding
ENSG00000126267	COX6B1	19:36139124-36149763	protein_coding

ENSG00000126883	NUP214	9:134000947-134110057	protein_coding
ENSG00000127022	CANX	5:179105628-179157926	protein_coding
ENSG00000127483	HP1BP3	1:21069153-21113816	protein_coding
ENSG00000127824	TUBA4A	2:220114432-220142892	protein_coding
ENSG00000128016	ZFP36	19:39897452-39900052	protein_coding
ENSG00000128340	RAC2	22:37621300-37640488	protein_coding
ENSG00000129353	SLC44A2	19:10713132-10755235	protein_coding
ENSG00000129657	SEC14L1	17:75082797-75213179	protein_coding
ENSG00000130066	SAT1	X:23801289-23804343	protein_coding
ENSG00000130429	ARPC1B	7:98971871-98992424	protein_coding
ENSG00000130592	LSP1	11:1874199-1913497	protein_coding
ENSG00000130755	GMFG	19:39818992-39833012	protein_coding
ENSG00000130775	THEMIS2	1:28199054-28213196	protein_coding
ENSG00000131051	RBM39	20:34291530-34330234	protein_coding
ENSG00000131171	SH3BGRL	X:80457441-80554046	protein_coding
ENSG00000131236	CAP1	1:40505904-40538321	protein_coding
ENSG00000131389	SLC6A6	3:14444075-14530857	protein_coding
ENSG00000131469	RPL27	17:41150289-41154976	protein_coding
ENSG00000132471	WBP2	17:73841779-73852588	protein_coding
ENSG00000132475	H3F3B	17:73772514-73781974	protein_coding
ENSG00000132510	KDM6B	17:7743221-7758114	protein_coding
ENSG00000132589	FLOT2	17:27206352-27224697	protein_coding
ENSG00000132824	SERINC3	20:43124861-43150750	protein_coding
ENSG00000132965	ALOX5AP	13:31309644-31338556	protein_coding
ENSG00000133112	TPT1	13:45911007-45915505	protein_coding
ENSG00000133606	MKRN1	7:140152839-140179369	protein_coding
ENSG00000133872	TMEM66	8:29920527-29940723	protein_coding
ENSG00000133961	NUMB	14:73741814-73930348	protein_coding
ENSG00000134294	SLC38A2	12:46751971-46766650	protein_coding
ENSG00000134419	RPS15A	16:18792616-18801705	protein_coding
ENSG00000134851	TMEM165	4:56262123-56319564	protein_coding
ENSG00000135655	USP15	12:62654118-62811211	protein_coding

ENSG00000135821	GLUL	1:182350838-182361341	protein_coding
ENSG00000135899	SP110	2:231032008-231090444	protein_coding
ENSG00000136156	ITM2B	13:48807293-48837063	protein_coding
ENSG00000136167	LCP1	13:46700054-46786006	protein_coding
ENSG00000136560	TANK	2:161993418-162092732	protein_coding
ENSG00000137154	RPS6	9:19375712-19380252	protein_coding
ENSG00000137207	YIPF3	6:43479564-43484728	protein_coding
ENSG00000137845	ADAM10	15:58887402-59042177	protein_coding
ENSG00000138293	NCOA4	10:51565107-51590734	protein_coding
ENSG00000139318	DUSP6	12:89741008-89747048	protein_coding
ENSG00000139436	GIT2	12:110367606-110434194	protein_coding
ENSG00000140199	SLC12A6	15:34525459-34630261	protein_coding
ENSG00000140264	SERF2	15:44069284-44094787	protein_coding
ENSG00000140455	USP3	15:63796792-63886839	protein_coding
ENSG00000140497	SCAMP2	15:75136070-75165706	protein_coding
ENSG00000140575	IQGAP1	15:90931449-91045475	protein_coding
ENSG00000140678	ITGAX	16:31366454-31394318	protein_coding
ENSG00000140853	NLRC5	16:57023396-57117443	protein_coding
ENSG00000141279	NPEPPS	17:45600307-45700642	protein_coding
ENSG00000141298	SSH2	17:27952955-28257294	protein_coding
ENSG00000141480	ARRB2	17:4613783-4624794	protein_coding
ENSG00000142347	MYO1F	19:8585673-8642461	protein_coding
ENSG00000142534	RPS11	19:49999621-50002946	protein_coding
ENSG00000142634	EFHD2	1:15736390-15756839	protein_coding
ENSG00000142657	PGD	1:10458648-10480201	protein_coding
ENSG00000142669	SH3BGRL3	1:26605666-26608007	protein_coding
ENSG00000142676	RPL11	1:24018268-24022915	protein_coding
ENSG00000142937	RPS8	1:45240922-45244451	protein_coding
ENSG00000143119	CD53	1:111415774-111442550	protein_coding
ENSG00000143384	MCL1	1:150547031-150552066	protein_coding
ENSG00000143409	FAM63A	1:150969024-150980851	protein_coding
ENSG00000143546	S100A8	1:153362507-153363664	protein_coding

ENSG00000143549	TPM3	1:154127783-154167124	protein_coding
ENSG00000143761	ARF1	1:228270360-228286912	protein_coding
ENSG00000143774	GUK1	1:228327662-228336685	protein_coding
ENSG00000145335	SNCA	4:90645249-90759466	protein_coding
ENSG00000145495	MARCH6	5:10353814-10440500	protein_coding
ENSG00000145592	RPL37	5:40825363-40835437	protein_coding
ENSG00000146112	PPP1R18	6:30644165-30655672	protein_coding
ENSG00000146278	PNRC1	6:89790469-89794879	protein_coding
ENSG00000147065	MSN	X:64808256-64961791	protein_coding
ENSG00000147162	OGT	X:70752932-70795747	protein_coding
ENSG00000147168	IL2RG	X:70327253-70331958	protein_coding
ENSG00000147416	ATP6V1B2	8:20054877-20084330	protein_coding
ENSG00000147454	SLC25A37	8:23386317-23432976	protein_coding
ENSG00000148700	ADD3	10:111756125-111895323	protein_coding
ENSG00000149273	RPS3	11:75110529-75133324	protein_coding
ENSG00000149311	ATM	11:108093210-108239829	protein_coding
ENSG00000149806	FAU	11:64888099-64889945	protein_coding
ENSG00000149925	ALDOA	16:30064410-30081778	protein_coding
ENSG00000150991	UBC	12:125396149-125401914	protein_coding
ENSG00000151702	FLI1	11:128556429-128683162	protein_coding
ENSG00000151883	PARP8	5:49961732-50142356	protein_coding
ENSG00000152601	MBNL1	3:151961616-152183569	protein_coding
ENSG00000153187	HNRNPU	1:245014467-245027844	protein_coding
ENSG00000153827	TRIP12	2:230628553-230787955	protein_coding
ENSG00000155926	SLA	8:134048972-134115298	protein_coding
ENSG00000155957	TMBIM4	12:66517708-66563852	protein_coding
ENSG00000156482	RPL30	8:99037078-99058697	protein_coding
ENSG00000156508	EEF1A1	6:74225472-74233520	protein_coding
ENSG00000157514	TSC22D3	X:106956450-107020572	protein_coding
ENSG00000158517	NCF1	7:74188308-74203659	protein_coding
ENSG00000158578	ALAS2	X:55035487-55057497	protein_coding
ENSG00000158710	TAGLN2	1:159887896-159895522	protein_coding

ENSG00000158856	DMTN	8:21906505-21940038	protein_coding
ENSG00000158869	FCER1G	1:161185023-161190489	protein_coding
ENSG00000159023	EPB41	1:29213602-29446553	protein_coding
ENSG00000159314	ARHGAP27	17:43471274-43511787	protein_coding
ENSG00000159346	ADIPOR1	1:202909950-202927700	protein_coding
ENSG00000159377	PSMB4	1:151372009-151374420	protein_coding
ENSG00000159592	GPBP1L1	1:46092975-46153785	protein_coding
ENSG00000159720	ATP6V0D1	16:67471916-67515140	protein_coding
ENSG00000160255	ITGB2	21:46305867-46351904	protein_coding
ENSG00000160410	SHKBP1	19:41082756-41097305	protein_coding
ENSG00000160593	AMICA1	11:118064454-118095809	protein_coding
ENSG00000161011	SQSTM1	5:179233387-179265078	protein_coding
ENSG00000162434	JAK1	1:65298911-65432187	protein_coding
ENSG00000162511	LAPTM5	1:31205315-31230667	protein_coding
ENSG00000162704	ARPC5	1:183592400-183604892	protein_coding
ENSG00000163041	H3F3A	1:226249551-226259702	protein_coding
ENSG00000163131	CTSS	1:150702671-150738433	protein_coding
ENSG00000163162	RNF149	2:101887680-101925163	protein_coding
ENSG00000163191	S100A11	1:152004981-152020383	protein_coding
ENSG00000163219	ARHGAP25	2:68906732-69053965	protein_coding
ENSG00000163220	S100A9	1:153330329-153333503	protein_coding
ENSG00000163466	ARPC2	2:219081816-219119079	protein_coding
ENSG00000163660	CCNL1	3:156864296-156878549	protein_coding
ENSG00000163736	PPBP	4:74852754-74853914	protein_coding
ENSG00000163931	TKT	3:53258722-53290068	protein_coding
ENSG00000164054	SHISA5	3:48509196-48542259	protein_coding
ENSG00000164329	PAPD4	5:78907942-78982471	protein_coding
ENSG00000164587	RPS14	5:149822752-149829319	protein_coding
ENSG00000164733	CTSB	8:11700032-11726957	protein_coding
ENSG00000164924	YWHAZ	8:101928752-101965616	protein_coding
ENSG00000165071	TMEM71	8:133697252-133772958	protein_coding
ENSG00000165406	MARCH8	10:45950034-46090354	protein_coding

ENSG00000166046	TCP11L2	12:106695706-106740793	protein_coding
ENSG00000166340	TPP1	11:6633999-6640692	protein_coding
ENSG00000166579	NDEL1	17:8316448-8393729	protein_coding
ENSG00000166710	B2M	15:45003674-45011075	protein_coding
ENSG00000166783	KIAA0430	16:15688242-15737023	protein_coding
ENSG00000166825	ANPEP	15:90328119-90358633	protein_coding
ENSG00000166888	STAT6	12:57489190-57525922	protein_coding
ENSG00000167173	C15orf39	15:75487983-75504510	protein_coding
ENSG00000167526	RPL13	16:89627064-89630950	protein_coding
ENSG00000167552	TUBA1A	12:49578578-49583107	protein_coding
ENSG00000167553	TUBA1C	12:49582518-49667114	protein_coding
ENSG00000167615	LENG8	19:54960064-54973217	protein_coding
ENSG00000167658	EEF2	19:3976053-3985467	protein_coding
ENSG00000167978	SRRM2	16:2802329-2822539	protein_coding
ENSG00000167996	FTH1	11:61727189-61735132	protein_coding
ENSG00000168010	ATG16L2	11:72525352-72554719	protein_coding
ENSG00000168066	SF1	11:64532077-64546258	protein_coding
ENSG00000168298	HIST1H1E	6:26156558-26157343	protein_coding
ENSG00000168310	IRF2	4:185308866-185395734	protein_coding
ENSG00000168394	TAP1	6:32812985-32821755	protein_coding
ENSG00000169045	HNRNPH1	5:179041178-179061785	protein_coding
ENSG00000169100	SLC25A6	X:1505044-1511617	protein_coding
ENSG00000169180	XPO6	16:28109299-28223241	protein_coding
ENSG00000169442	CD52	1:26644447-26647014	protein_coding
ENSG00000169554	ZEB2	2:145141647-145282147	protein_coding
ENSG00000169564	PCBP1	2:70314584-70316332	protein_coding
ENSG00000169896	ITGAM	16:31271310-31344213	protein_coding
ENSG00000170248	PDCD6IP	3:33839843-33911194	protein_coding
ENSG00000170296	GABARAP	17:7143332-7146089	protein_coding
ENSG00000170315	UBB	17:16284111-16286059	protein_coding
ENSG00000170776	AKAP13	15:85923801-86292586	protein_coding
ENSG00000170889	RPS9	19:54704609-54752862	protein_coding

ENSG00000171051	FPR1	19:52248424-52307363	protein_coding
ENSG00000171552	BCL2L1	20:30252254-30311792	protein_coding
ENSG00000172081	MOB3A	19:2071036-2096672	protein_coding
ENSG00000172270	BSG	19:571296-583493	protein_coding
ENSG00000172493	AFF1	4:87856153-88062206	protein_coding
ENSG00000172757	CFL1	11:65590492-65629497	protein_coding
ENSG00000172795	DCP2	5:112312398-112356667	protein_coding
ENSG00000172809	RPL38	17:72199720-72206794	protein_coding
ENSG00000172936	MYD88	3:38179968-38184513	protein_coding
ENSG00000173559	NABP1	2:192542793-192561385	protein_coding
ENSG00000173575	CHD2	15:93426525-93571237	protein_coding
ENSG00000173812	EIF1	17:39845136-39848920	protein_coding
ENSG00000173889	PHC3	3:169804519-169899537	protein_coding
ENSG00000174444	RPL4	15:66790354-66816870	protein_coding
ENSG00000174748	RPL15	3:23958035-23965183	protein_coding
ENSG00000175215	CTDSP2	12:58213709-58240522	protein_coding
ENSG00000175567	UCP2	11:73685711-73694352	protein_coding
ENSG00000177105	RHOG	11:3848207-3862213	protein_coding
ENSG00000177156	TALDO1	11:747328-765024	protein_coding
ENSG00000177600	RPLP2	11:809646-812880	protein_coding
ENSG00000177663	IL17RA	22:17565843-17596583	protein_coding
ENSG00000177885	GRB2	17:73314156-73401790	protein_coding
ENSG00000177954	RPS27	1:153963234-153964626	protein_coding
ENSG00000178719	GRINA	8:145064225-145067583	protein_coding
ENSG00000178927	C17orf62	17:80400464-80408705	protein_coding
ENSG00000179820	MYADM	19:54369476-54379691	protein_coding
ENSG00000180353	HCLS1	3:121350245-121379774	protein_coding
ENSG00000180573	HIST1H2AC	6:26124372-26139344	protein_coding
ENSG00000180596	HIST1H2BC	6:26115100-26124154	protein_coding
ENSG00000180871	CXCR2	2:218990011-219001976	protein_coding
ENSG00000181274	FRAT2	10:99092254-99094458	protein_coding
ENSG00000181788	SIAH2	3:150458913-150481264	protein_coding

ENSG00000182578	CSF1R	5:149432853-149492935	protein_coding
ENSG00000182899	RPL35A	3:197676857-197683481	protein_coding
ENSG00000183283	DAZAP2	12:51632075-51665146	protein_coding
ENSG00000183486	MX2	21:42733869-42781317	protein_coding
ENSG00000183508	FAM46C	1:118148555-118170994	protein_coding
ENSG00000184007	PTP4A2	1:32372021-32410457	protein_coding
ENSG00000184009	ACTG1	17:79476996-79490873	protein_coding
ENSG00000184922	FMNL1	17:43298810-43324687	protein_coding
ENSG00000185201	IFITM2	11:307630-315272	protein_coding
ENSG00000185215	TNFAIP2	14:103589778-103603776	protein_coding
ENSG00000185236	RAB11B	19:8454864-8469318	protein_coding
ENSG00000185650	ZFP36L1	14:69254376-69263190	protein_coding
ENSG00000185811	IKZF1	7:50343719-50472799	protein_coding
ENSG00000185864	NPIPB4	16:21845889-21892148	protein_coding
ENSG00000185883	ATP6V0C	16:2563870-2570219	protein_coding
ENSG00000185905	C16orf54	16:29753783-29757327	protein_coding
ENSG00000187446	CHP1	15:41523036-41574043	protein_coding
ENSG00000187764	SEMA4D	9:91975701-92113045	protein_coding
ENSG00000188186	LAMTOR4	7:99746529-99753567	protein_coding
ENSG00000188404	SELL	1:169659807-169680839	protein_coding
ENSG00000196126	HLA-DRB1	6:32546545-32557625	protein_coding
ENSG00000196154	S100A4	1:153516088-153522612	protein_coding
ENSG00000196352	CD55	1:207494852-207534311	protein_coding
ENSG00000196405	EVL	14:100437785-100610573	protein_coding
ENSG00000196531	NACA	12:57106211-57125412	protein_coding
ENSG00000197081	IGF2R	6:160390130-160534539	protein_coding
ENSG00000197111	PCBP2	12:53835524-53874946	protein_coding
ENSG00000197249	SERPINA1	14:94843083-94857030	protein_coding
ENSG00000197324	LRP10	14:23340821-23350789	protein_coding
ENSG00000197448	GSTK1	7:142941185-142967947	protein_coding
ENSG00000197548	ATG7	3:11313994-11599139	protein_coding
ENSG00000197622	CDC42SE1	1:151023446-151042801	protein_coding

ENSG00000197629	MPEG1	11:58975982-58980424	protein_coding
ENSG00000197746	PSAP	10:73576054-73611126	protein_coding
ENSG00000197756	RPL37A	2:217362911-217443903	protein_coding
ENSG00000197956	S100A6	1:153507074-153508720	protein_coding
ENSG00000197971	MBP	18:74690782-74845639	protein_coding
ENSG00000198408	MGEA5	10:103544199-103578696	protein_coding
ENSG00000198563	DDX39B	6:31497995-31510225	protein_coding
ENSG00000198625	MDM4	1:204485510-204542871	protein_coding
ENSG00000198668	CALM1	14:90862845-90874605	protein_coding
ENSG00000198712	MT-CO2	MT:7585-8269	protein_coding
ENSG00000198727	MT-CYB	MT:14746-15887	protein_coding
ENSG00000198755	RPL10A	6:35436184-35438562	protein_coding
ENSG00000198763	MT-ND2	MT:4469-5511	protein_coding
ENSG00000198786	MT-ND5	MT:12336-14148	protein_coding
ENSG00000198804	MT-CO1	MT:5903-7445	protein_coding
ENSG00000198837	DENND4B	1:153901976-153919172	protein_coding
ENSG00000198840	MT-ND3	MT:10058-10404	protein_coding
ENSG00000198851	CD3E	11:118175259-118186890	protein_coding
ENSG00000198858	R3HDM4	19:896502-913240	protein_coding
ENSG00000198876	DCAF12	9:34086384-34127397	protein_coding
ENSG00000198886	MT-ND4	MT:10759-12137	protein_coding
ENSG00000198888	MT-ND1	MT:3306-4262	protein_coding
ENSG00000198938	MT-CO3	MT:9206-9990	protein_coding
ENSG00000204160	ZDHHC18	1:27153200-27184093	protein_coding
ENSG00000204287	HLA-DRA	6:32407618-32412823	protein_coding
ENSG00000204463	BAG6	6:31606804-31620482	protein_coding
ENSG00000204472	AIF1	6:31582960-31584798	protein_coding
ENSG00000204592	HLA-E	6:30457243-30461982	protein_coding
ENSG00000204628	GNB2L1	5:180663908-180675096	protein_coding
ENSG00000204642	HLA-F	6:29690551-29706305	protein_coding
ENSG00000205352	PRR13	12:53835388-53840429	protein_coding
ENSG00000205542	TMSB4X	X:12993226-12995346	protein_coding

ENSG00000206503	HLA-A	6:29909036-29913661	protein_coding
ENSG00000212907	MT-ND4L	MT:10469-10766	protein_coding
ENSG00000213145	CRIP1	14:105952653-105955284	protein_coding
ENSG00000213240	NOTCH2NL	1:145209118-145291972	protein_coding
ENSG00000213719	CLIC1	6:31698357-31707540	protein_coding
ENSG00000213928	IRF9	14:24630261-24635774	protein_coding
ENSG00000213983	AP1G2	14:24028773-24037279	protein_coding
ENSG00000214078	CPNE1	20:34213952-34252878	protein_coding
ENSG00000215301	DDX3X	X:41192650-41223725	protein_coding
ENSG00000216490	IFI30	19:18283971-18288927	protein_coding
ENSG00000221983	UBA52	19:18682539-18688360	protein_coding
ENSG00000227507	LTB	6:31548301-31550299	protein_coding
ENSG00000228253	MT-ATP8	MT:8365-8572	protein_coding
ENSG00000228474	OST4	2:27293339-27294641	protein_coding
ENSG00000231389	HLA-DPA1	6:33032345-33048552	protein_coding
ENSG00000231925	TAPBP	6:33267470-33282164	protein_coding
ENSG00000234745	HLA-B	6:31321648-31324965	protein_coding
ENSG00000240065	PSMB9	6:32811912-32827362	protein_coding
ENSG00000241553	ARPC4	3:9834178-9849410	protein_coding
ENSG00000243716	NPIPB5	16:22490441-22547842	protein_coding
ENSG00000255823	MTRNR2L8	11:10529433-10530723	protein_coding
ENSG00000269028	MTRNR2L12	3:96335980-96337000	protein_coding

Supplemental Table 4. GO analysis of 435 protein coding genes

Category	Term	Count	%	PValue	Fold Enrichment	Benjamini_FDR
GOTERM_BP_FAT	GO:0006414~translational elongation	41	10.25	1.32E-37	15.78036	2.64E-34
SP_PIR_KEYWORDS	ribosome	34	8.5	2.03E-36	22.45305	7.88E-34
SP_PIR_KEYWORDS	acetylation	151	37.75	7.58E-34	2.762585	1.47E-31
GOTERM_CC_FAT	GO:0022626~cytosolic ribosome	34	8.5	9.95E-32	16.35757	3.40E-29
SP_PIR_KEYWORDS	phosphoprotein	259	64.75	1.58E-28	1.719107	2.04E-26
SP_PIR_KEYWORDS	ribosomal protein	38	9.5	1.14E-25	9.744174	1.11E-23
SP_PIR_KEYWORDS	ribonucleoprotein	44	11	3.53E-25	7.602699	2.75E-23
KEGG_PATHWAY	hsa03010:Ribosome	35	8.75	2.39E-25	9.559297	2.77E-23
SP_PIR_KEYWORDS	protein biosynthesis	37	9.25	1.43E-24	9.487749	9.26E-23
GOTERM_CC_FAT	GO:0033279~ribosomal subunit	33	8.25	3.80E-23	10.04683	6.50E-21
GOTERM_MF_FAT	GO:0003735~structural constituent of ribosome	37	9.25	3.31E-23	8.459619	1.84E-20
GOTERM_CC_FAT	GO:0005840~ribosome	40	10	1.62E-22	7.250142	1.85E-20
GOTERM_CC_FAT	GO:0022627~cytosolic small ribosomal subunit	21	5.25	6.85E-22	20.45899	5.86E-20
GOTERM_CC_FAT	GO:0044445~cytosolic part	34	8.5	9.46E-22	8.716865	6.47E-20
GOTERM_CC_FAT	GO:0030529~ribonucleoprotein complex	55	13.75	2.92E-19	4.161793	1.66E-17
GOTERM_CC_FAT	GO:0005829~cytosol	90	22.5	2.83E-18	2.637035	1.38E-16
GOTERM_BP_FAT	GO:0006412~translation	44	11	7.76E-19	5.167483	7.78E-16
GOTERM_CC_FAT	GO:0015935~small ribosomal subunit	21	5.25	3.82E-17	12.98984	1.63E-15
SP_PIR_KEYWORDS	heterodimer	20	5	3.27E-13	9.360781	1.82E-11
GOTERM_MF_FAT	GO:0005198~structural molecule activity	53	13.25	1.03E-13	3.211034	2.87E-11
GOTERM_CC_FAT	GO:0043228~non-membrane-bounded organelle	121	30.25	2.89E-12	1.816376	1.10E-10
GOTERM_CC_FAT	GO:0043232~intracellular non-membrane-bounded organelle	121	30.25	2.89E-12	1.816376	1.10E-10
GOTERM_MF_FAT	GO:0003723~RNA binding	53	13.25	1.28E-11	2.83537	2.37E-09
SP_PIR_KEYWORDS	cytoplasm	122	30.5	7.13E-11	1.76512	3.47E-09
GOTERM_CC_FAT	GO:0022625~cytosolic large ribosomal subunit	12	3	1.69E-09	12.30616	5.77E-08
SP_PIR_KEYWORDS	host-virus interaction	26	6.5	1.39E-09	4.397925	6.01E-08
SP_PIR_KEYWORDS	actin-binding	24	6	2.03E-09	4.68418	7.90E-08
GOTERM_CC_FAT	GO:0015629~actin cytoskeleton	27	6.75	5.79E-09	3.911438	1.80E-07

GOTERM_BP_FAT	GO:0048002~antigen processing and presentation of peptide antigen	11	2.75	9.43E-10	15.27176	6.30E-07
SP_PIR_KEYWORDS	transmembrane protein	38	9.5	2.11E-08	2.853434	7.45E-07
GOTERM_MF_FAT	GO:0003779~actin binding	30	7.5	7.06E-09	3.534777	9.82E-07
GOTERM_BP_FAT	GO:0030029~actin filament-based process	26	6.5	2.96E-09	4.193828	1.48E-06
GOTERM_BP_FAT	GO:0030036~actin cytoskeleton organization	25	6.25	3.86E-09	4.300173	1.55E-06
GOTERM_BP_FAT	GO:0006955~immune response	46	11.5	7.09E-09	2.591571	2.37E-06
SP_PIR_KEYWORDS	methylation	21	5.25	1.68E-07	4.183341	5.44E-06
GOTERM_BP_FAT	GO:0006952~defense response	42	10.5	1.91E-08	2.65478	5.46E-06
GOTERM_BP_FAT	GO:0019882~antigen processing and presentation	15	3.75	2.19E-08	7.025343	5.49E-06
SP_PIR_KEYWORDS	nucleotide binding	14	3.5	3.27E-07	6.307591	9.79E-06
SP_PIR_KEYWORDS	EF hand	11	2.75	3.85E-07	8.838137	1.07E-05
GOTERM_MF_FAT	GO:0008092~cytoskeletal protein binding	36	9	1.10E-07	2.74366	1.22E-05
KEGG_PATHWAY	hsa04612:Antigen processing and presentation	17	4.25	2.19E-07	4.866851	1.27E-05
SP_PIR_KEYWORDS	P-loop	14	3.5	8.40E-07	5.818209	2.18E-05
GOTERM_CC_FAT	GO:0015934~large ribosomal subunit	12	3	9.29E-07	6.979614	2.65E-05
SP_PIR_KEYWORDS	cytoskeleton	34	8.5	1.33E-06	2.577158	3.24E-05
SP_PIR_KEYWORDS	ubl conjugation	32	8	2.04E-06	2.623566	4.66E-05
GOTERM_CC_FAT	GO:0031252~cell leading edge	16	4	2.42E-06	4.518204	6.38E-05
SP_PIR_KEYWORDS	blocked amino end	12	3	3.10E-06	6.288003	6.71E-05
KEGG_PATHWAY	hsa05130:Pathogenic Escherichia coli infection	13	3.25	2.86E-06	5.419331	1.11E-04
SP_PIR_KEYWORDS	duplication	18	4.5	5.74E-06	3.805896	1.17E-04
GOTERM_BP_FAT	GO:0019884~antigen processing and presentation of exogenous antigen	7	1.75	6.89E-07	19.43678	1.53E-04
KEGG_PATHWAY	hsa05416:Viral myocarditis	14	3.5	5.72E-06	4.685402	1.66E-04
GOTERM_CC_FAT	GO:0005773~vacuole	21	5.25	7.53E-06	3.247459	1.84E-04
UP_SEQ_FEATURE	mutagenesis site	78	19.5	1.74E-07	1.827079	2.20E-04
SP_PIR_KEYWORDS	isopeptide bond	21	5.25	1.20E-05	3.173569	2.33E-04
GOTERM_CC_FAT	GO:0005885~Arp2/3 protein complex	5	1.25	1.39E-05	27.83537	3.16E-04
GOTERM_BP_FAT	GO:0006928~cell motion	32	8	1.80E-06	2.618851	3.60E-04
SP_PIR_KEYWORDS	rna-binding	28	7	2.41E-05	2.499675	4.46E-04
SP_PIR_KEYWORDS	mhc i	5	1.25	3.44E-05	24.10401	6.07E-04

GOTERM_BP_FAT	GO:0002478~antigen processing and presentation of exogenous peptide antigen	6	1.5	4.39E-06	21.20376	7.33E-04
GOTERM_BP_FAT	GO:0045321~leukocyte activation	21	5.25	4.33E-06	3.373326	7.88E-04
UP_SEQ_FEATURE	domain:Ig-like C1-type	9	2.25	1.41E-06	10.77801	8.94E-04
SP_PIR_KEYWORDS	calcium binding	11	2.75	5.48E-05	5.148429	9.26E-04
SP_PIR_KEYWORDS	immune response	16	4	7.02E-05	3.44343	0.001138
GOTERM_BP_FAT	GO:0007010~cytoskeleton organization	29	7.25	7.95E-06	2.585627	0.001224
GOTERM_CC_FAT	GO:0031988~membrane-bounded vesicle	32	8	5.78E-05	2.195465	0.001235
GOTERM_BP_FAT	GO:0045730~respiratory burst	6	1.5	1.17E-05	17.94164	0.001676
GOTERM_CC_FAT	GO:0000323~lytic vacuole	17	4.25	1.05E-04	3.139724	0.00212
GOTERM_CC_FAT	GO:0005764~lysosome	17	4.25	1.05E-04	3.139724	0.00212
GOTERM_BP_FAT	GO:0001775~cell activation	22	5.5	1.65E-05	2.979855	0.002197
GOTERM_CC_FAT	GO:0005856~cytoskeleton	58	14.5	1.72E-04	1.636663	0.003271
SP_PIR_KEYWORDS	RNA binding	6	1.5	2.45E-04	10.33029	0.003813
GOTERM_CC_FAT	GO:0031982~vesicle	34	8.5	2.32E-04	1.977557	0.003965
SMART	SM00407:IGc1	10	2.5	2.60E-05	6.268296	0.003968
GOTERM_CC_FAT	GO:0030863~cortical cytoskeleton	8	2	2.29E-04	6.362369	0.004121
SP_PIR_KEYWORDS	diamond-blackfan anemia	4	1	2.89E-04	27.54744	0.004319
SP_PIR_KEYWORDS	Proto-oncogene	15	3.75	3.27E-04	3.144001	0.0047
SP_PIR_KEYWORDS	rrna-binding	5	1.25	3.47E-04	14.17883	0.004811
KEGG_PATHWAY	hsa04810:Regulation of actin cytoskeleton	22	5.5	2.19E-04	2.431428	0.005061
GOTERM_CC_FAT	GO:0033179~proton-transporting V-type ATPase, V0 domain	4	1	3.13E-04	25.97967	0.005091
SP_PIR_KEYWORDS	Hydrogen ion transport	7	1.75	4.45E-04	7.030336	0.005952
GOTERM_BP_FAT	GO:0002474~antigen processing and presentation of peptide antigen via MHC class I	6	1.5	5.18E-05	13.72008	0.006085
GOTERM_BP_FAT	GO:0034621~cellular macromolecular complex subunit organization	24	6	4.91E-05	2.613349	0.00613
SP_PIR_KEYWORDS	ADP-ribosylation	6	1.5	5.45E-04	8.765095	0.007038
SP_PIR_KEYWORDS	methylated amino acid	5	1.25	6.73E-04	12.05201	0.008408
GOTERM_CC_FAT	GO:0005884~actin filament	7	1.75	5.66E-04	6.653331	0.008755
GOTERM_CC_FAT	GO:0042611~MHC protein complex	8	2	5.93E-04	5.469405	0.00878
GOTERM_CC_FAT	GO:0030864~cortical actin cytoskeleton	6	1.5	6.43E-04	8.35061	0.009131

GOTERM_BP_FAT	GO:0007015~actin filament organization	10	2.5	8.88E-05	5.399106	0.00984
SP_PIR_KEYWORDS	thiolester bond	6	1.5	8.22E-04	8.03467	0.009948
GOTERM_BP_FAT	GO:0042110~T cell activation	13	3.25	9.49E-05	4.010764	0.009956
SP_PIR_KEYWORDS	Spliceosome	10	2.5	8.81E-04	4.017335	0.010332
BIOCARTA	h_mhcPathway:Antigen Processing and Presentation	6	1.5	7.44E-05	11.1974	0.012123
GOTERM_BP_FAT	GO:0042274~ribosomal small subunit biogenesis	5	1.25	1.22E-04	17.6698	0.012124
SP_PIR_KEYWORDS	acetylated amino end	9	2.25	0.001111	4.338722	0.012282
GOTERM_CC_FAT	GO:0016469~proton-transporting two-sector ATPase complex	7	1.75	9.40E-04	6.061924	0.012298
SP_PIR_KEYWORDS	lysosome	11	2.75	0.001096	3.558981	0.012468
GOTERM_CC_FAT	GO:0016023~cytoplasmic membrane-bounded vesicle	28	7	9.23E-04	1.983902	0.012551
SP_PIR_KEYWORDS	actin binding	6	1.5	0.001341	7.231203	0.014397
GOTERM_BP_FAT	GO:0002274~myeloid leukocyte activation	8	2	1.56E-04	6.76062	0.014735
GOTERM_BP_FAT	GO:0002252~immune effector process	13	3.25	1.71E-04	3.771316	0.015453
GOTERM_CC_FAT	GO:0043020~NADPH oxidase complex	4	1	0.001243	17.31978	0.015627
SP_PIR_KEYWORDS	surface antigen	6	1.5	0.001676	6.88686	0.017483
GOTERM_CC_FAT	GO:0033176~proton-transporting V-type ATPase complex	5	1.25	0.001473	9.742378	0.017844
GOTERM_BP_FAT	GO:0034622~cellular macromolecular complex assembly	21	5.25	2.11E-04	2.567122	0.018205
GOTERM_CC_FAT	GO:0001726~ruffle	8	2	0.001569	4.653076	0.018347
INTERPRO	IPR003597:Immunoglobulin C1-set	10	2.5	2.38E-05	6.396483	0.018868
UP_SEQ_FEATURE	region of interest:Alpha-1	6	1.5	4.57E-05	14.37068	0.0191
UP_SEQ_FEATURE	region of interest:Alpha-2	6	1.5	4.57E-05	14.37068	0.0191
GOTERM_BP_FAT	GO:0034220~ion transmembrane transport	8	2	2.34E-04	6.346704	0.019329
KEGG_PATHWAY	hsa04670:Leukocyte transendothelial migration	14	3.5	0.001196	2.819183	0.019631
KEGG_PATHWAY	hsa04514:Cell adhesion molecules (CAMs)	15	3.75	0.001136	2.700191	0.021735
GOTERM_CC_FAT	GO:0048770~pigment granule	9	2.25	0.001969	3.940737	0.022216
GOTERM_CC_FAT	GO:0042470~melanosome	9	2.25	0.001969	3.940737	0.022216
GOTERM_BP_FAT	GO:0006916~anti-apoptosis	16	4	2.82E-04	3.019306	0.022319
SP_PIR_KEYWORDS	chronic granulomatous disease	3	0.75	0.002493	36.15602	0.025226
GOTERM_BP_FAT	GO:0002443~leukocyte mediated immunity	10	2.5	3.50E-04	4.520182	0.026658
GOTERM_MF_FAT	GO:0003729~mRNA binding	9	2.25	2.93E-04	5.237897	0.026833
GOTERM_BP_FAT	GO:0002684~positive regulation of immune system process	17	4.25	4.36E-04	2.776683	0.029675

KEGG_PATHWAY	hsa05110:Vibrio cholerae infection	9	2.25	0.00208	3.818842	0.029738
GOTERM_BP_FAT	GO:0032956~regulation of actin cytoskeleton organization	10	2.5	4.53E-04	4.367816	0.029812
GOTERM_BP_FAT	GO:0008219~cell death	35	8.75	4.26E-04	1.892315	0.030019
GOTERM_BP_FAT	GO:0016265~death	35	8.75	4.74E-04	1.879247	0.030196
GOTERM_BP_FAT	GO:0043122~regulation of I-kappaB kinase/NF-kappaB cascade	11	2.75	4.19E-04	3.996348	0.030655
GOTERM_CC_FAT	GO:0005887~integral to plasma membrane	47	11.75	0.002829	1.541723	0.030773
GOTERM_BP_FAT	GO:0015985~energy coupled proton transport, down electrochemical gradient	7	1.75	5.01E-04	6.802874	0.0309
GOTERM_BP_FAT	GO:0015986~ATP synthesis coupled proton transport	7	1.75	5.01E-04	6.802874	0.0309
GOTERM_CC_FAT	GO:0033177~proton-transporting two-sector ATPase complex, proton-transporting domain	5	1.25	0.002981	8.118648	0.0314
GOTERM_MF_FAT	GO:0003924~GTPase activity	16	4	4.07E-04	2.912701	0.031825
GOTERM_BP_FAT	GO:0022604~regulation of cell morphogenesis	12	3	5.51E-04	3.560937	0.03291
GOTERM_BP_FAT	GO:0032970~regulation of actin filament-based process	10	2.5	5.79E-04	4.225387	0.033556
GOTERM_BP_FAT	GO:0050900~leukocyte migration	8	2	6.04E-04	5.455939	0.033984
BIOCARTA	h_neutrophilPathway:Neutrophil and Its Surface Molecules	5	1.25	4.31E-04	11.66396	0.034724
GOTERM_BP_FAT	GO:0007159~leukocyte adhesion	6	1.5	6.52E-04	8.330049	0.035658
SP_PIR_KEYWORDS	mhc ii	5	1.25	0.003652	7.775487	0.035832
GOTERM_CC_FAT	GO:0009986~cell surface	19	4.75	0.003734	2.127646	0.03803
UP_SEQ_FEATURE	region of interest:Connecting peptide	7	1.75	1.23E-04	8.8241	0.038248
KEGG_PATHWAY	hsa05330:Allograft rejection	7	1.75	0.003417	4.620327	0.038922
SP_PIR_KEYWORDS	polyprotein	3	0.75	0.004098	28.92481	0.039145
GOTERM_BP_FAT	GO:0006954~inflammatory response	20	5	7.45E-04	2.392219	0.039552
GOTERM_CC_FAT	GO:0031410~cytoplasmic vesicle	29	7.25	0.004137	1.760305	0.040838
GOTERM_CC_FAT	GO:0031226~intrinsic to plasma membrane	47	11.75	0.004401	1.507463	0.042183
KEGG_PATHWAY	hsa04142:Lysosome	13	3.25	0.003369	2.640187	0.042561
GOTERM_BP_FAT	GO:0002495~antigen processing and presentation of peptide antigen via MHC class II	4	1	8.51E-04	19.43678	0.042819
GOTERM_BP_FAT	GO:0019886~antigen processing and presentation of exogenous peptide antigen via MHC class II	4	1	8.51E-04	19.43678	0.042819

GOTERM_BP_FAT	GO:0034097~response to cytokine stimulus	9	2.25	9.24E-04	4.428634	0.043168
GOTERM_BP_FAT	GO:0008064~regulation of actin polymerization or depolymerization	8	2	9.13E-04	5.098172	0.043651
GOTERM_BP_FAT	GO:0015992~proton transport	8	2	9.13E-04	5.098172	0.043651
GOTERM_MF_FAT	GO:0005200~structural constituent of cytoskeleton	9	2.25	6.44E-04	4.671638	0.043758
GOTERM_BP_FAT	GO:0006915~apoptosis	30	7.5	8.50E-04	1.937221	0.043872
GOTERM_BP_FAT	GO:0016192~vesicle-mediated transport	29	7.25	8.99E-04	1.957176	0.044079
GOTERM_CC_FAT	GO:0042612~MHC class I protein complex	5	1.25	0.005301	6.958841	0.045537
GOTERM_CC_FAT	GO:0031967~organelle envelope	28	7	0.004936	1.759913	0.045917
GOTERM_CC_FAT	GO:0044448~cell cortex part	8	2	0.005298	3.756098	0.046682
GOTERM_CC_FAT	GO:0031975~envelope	28	7	0.005206	1.754255	0.047105
SP_PIR_KEYWORDS	membrane protein	7	1.75	0.00521	4.382547	0.04723
SP_PIR_KEYWORDS	proteoglycan	6	1.5	0.005093	5.356447	0.047289
GOTERM_BP_FAT	GO:0012501~programmed cell death	30	7.5	0.001065	1.908686	0.048433
GOTERM_BP_FAT	GO:0006818~hydrogen transport	8	2	0.001108	4.936325	0.04924
GOTERM_BP_FAT	GO:0030832~regulation of actin filament length	8	2	0.001108	4.936325	0.04924

Supplemental Table 5. LincRNAs highly expressed in whole blood (36 lincRNAs with log2 (FPKM) > 1 in all samples)

**gene_id**	**gene_short_name**	**locus**	**gene_biotype**
ENSG00000172965	MIR4435-1HG	2:111953926-112252677	lincRNA
ENSG00000188971	RP11-427H3.3	2:69685128-69693664	lincRNA
ENSG00000204261	TAPSAR1	6:32811862-32814272	lincRNA
ENSG00000214837	RP11-261C10.3	1:243242158-243265035	lincRNA
ENSG00000222041	LINC00152	2:87754886-87906324	lincRNA
ENSG00000223804	RP6-206I17.1	1:143647637-143745417	lincRNA
ENSG00000224397	RP11-290F20.3	20:48884022-48896332	lincRNA
ENSG00000225484	RP11-773D16.1	10:81563812-81586350	lincRNA
ENSG00000226328	CTA-217C2.1	22:45528900-45559662	lincRNA

ENSG00000228327	RP11-206L10.2	1:700236-714006	lincRNA
ENSG00000230590	FTX	X:73183789-73513409	lincRNA
ENSG00000230724	LINC01001	11:127114-139612	lincRNA
ENSG00000233013	FAM157B	9:141106636-141143444	lincRNA
ENSG00000235398	LINC00623	1:144299757-144341756	lincRNA
ENSG00000236438	FAM157A	3:197879236-197925886	lincRNA
ENSG00000237094	RP4-669L17.10	1:317719-453948	lincRNA
ENSG00000237943	PRKCQ-AS1	10:6622380-6633059	lincRNA
ENSG00000238035	AC138035.2	5:180733502-180755068	lincRNA
ENSG00000238113	RP11-262H14.1	9:66457284-66469310	lincRNA
ENSG00000240618	RP11-206L10.5	1:694411-700305	lincRNA
ENSG00000241163	LINC00877	3:72084450-72291716	lincRNA
ENSG00000244625	CTA-211A9.5	22:27068729-27176170	lincRNA
ENSG00000245164	LINC00861	8:126934551-126963493	lincRNA
ENSG00000246067	RAB30-AS1	11:82783107-82817761	lincRNA
ENSG00000248323	LUCAT1	5:90598845-90610219	lincRNA
ENSG00000259758	CASC7	8:141530254-141539600	lincRNA
ENSG00000260032	LINC00657	20:34633543-34638882	lincRNA
ENSG00000261804	RP11-44F14.2	16:53407404-53418657	lincRNA
ENSG00000262370	RP11-473M20.9	16:3126911-3137101	lincRNA
ENSG00000264112	RP11-159D12.2	17:56066399-56072211	lincRNA
ENSG00000267427	CTC-503J8.6	19:6210389-6212492	lincRNA
ENSG00000269614	RP11-277L2.5	1:149587501-149587988	lincRNA
ENSG00000269640	CTD-2521M24.9	19:17516502-17529713	lincRNA
ENSG00000269900	RMRP	9:35657747-35658015	lincRNA
ENSG00000270949	RP11-288H12.4	6:160529405-160532536	lincRNA
ENSG00000271204	RP11-138A9.1	7:130614967-130616965	lincRNA

Supplemental Table 6. Results of adjusting for additional 9 clinical covariates for 68 coding gene and 2 lincRNA signatures identified by Model 1.

				Model 1: adjusted for sex, batch, and two SVs			Model 2: adjusted for additional 9 covariates		
**gene_name**	**locus**	**gene_biotype**	**BaseMean**	**log2FD**	**pvalue**	**padj.BH**	**log2FD**	**pvalue**	**padj.BH**
**APOD**	3:195295572-195311076	protein_coding	301	**-0.194**	**1.29E-06**	**7.54E-03**	**-0.233**	**4.64E-07**	**2.80E-03**
**DUS1L**	17:80015381-80023763	protein_coding	902	**0.411**	**1.97E-06**	**7.54E-03**	**0.488**	**1.67E-07**	**2.02E-03**
**AFF3**	2:100162322-100759201	protein_coding	1478	**-0.444**	**2.12E-06**	**7.54E-03**	**-0.427**	**1.32E-05**	**4.74E-02**
**IRF7**	11:612552-615999	protein_coding	1652	**0.414**	**2.50E-06**	**7.54E-03**	**0.382**	**5.19E-05**	**7.15E-02**
IPO5	13:98605911-98676551	protein_coding	1866	-0.321	3.75E-06	9.05E-03	-0.281	3.43E-04	1.50E-01
SH3PXD2A	10:105348284-105615301	protein_coding	927	-0.405	5.93E-06	1.19E-02	-0.318	8.23E-04	2.07E-01
BACH2	6:90636247-91006627	protein_coding	3905	-0.368	1.09E-05	1.88E-02	-0.310	6.97E-04	2.04E-01
PAX5	9:36833271-37034103	protein_coding	1406	-0.408	1.67E-05	2.52E-02	-0.354	3.06E-04	1.50E-01
PHF6	X:133507282-133562820	protein_coding	704	-0.328	1.99E-05	2.67E-02	-0.317	2.20E-04	1.44E-01
FCRL2	1:157715522-157746922	protein_coding	868	-0.401	2.26E-05	2.72E-02	-0.349	3.47E-04	1.50E-01
VEZT	12:95611521-95696566	protein_coding	1024	-0.321	2.97E-05	3.01E-02	-0.287	7.96E-04	2.04E-01
**TRIM46**	1:155145872-155157447	protein_coding	159	0.390	3.52E-05	3.01E-02	0.415	1.97E-05	**4.74E-02**
IFI6	1:27992571-27998729	protein_coding	1711	0.390	3.60E-05	3.01E-02	0.377	1.14E-04	1.20E-01
RAD52	12:1021242-1099219	protein_coding	1090	-0.302	3.75E-05	3.01E-02	-0.284	5.90E-04	1.92E-01
BLK	8:11351509-11422113	protein_coding	562	-0.390	3.87E-05	3.01E-02	-0.334	6.87E-04	2.04E-01
HLA-F	6:29690551-29706305	protein_coding	5562	0.267	3.99E-05	3.01E-02	0.279	1.93E-04	1.44E-01
**IFI27**	14:94571181-94583033	protein_coding	170	0.318	4.54E-05	3.06E-02	0.340	4.48E-05	**7.15E-02**
HNRNPR	1:23630263-23670829	protein_coding	2946	-0.260	4.56E-05	3.06E-02	-0.223	2.30E-03	3.06E-01
ZNF44	19:12335500-12405702	protein_coding	671	-0.301	5.16E-05	3.19E-02	-0.257	2.05E-03	2.91E-01
FCER2	19:7753643-7767032	protein_coding	592	-0.379	5.29E-05	3.19E-02	-0.312	1.35E-03	2.48E-01
FRS2	12:69864128-69973562	protein_coding	1778	-0.256	5.88E-05	3.28E-02	-0.236	1.35E-03	2.48E-01
**HBG1**	11:5269312-5271122	protein_coding	17515	0.346	5.98E-05	3.28E-02	0.389	1.75E-05	**4.74E-02**
PRKDC	8:48685668-48872743	protein_coding	9155	-0.215	6.59E-05	3.37E-02	-0.246	1.19E-04	1.20E-01
MS4A1	11:60223224-60238233	protein_coding	3170	-0.375	6.91E-05	3.37E-02	-0.332	7.28E-04	2.04E-01

FCRLA	1:161676761-161684142	protein_coding	562	-0.372	6.98E-05	3.37E-02	-0.337	5.07E-04	1.82E-01
ACADVL	17:7120443-7128592	protein_coding	3648	0.263	8.06E-05	3.70E-02	0.247	1.15E-03	2.41E-01
CCDC141	2:179694483-179914813	protein_coding	703	-0.372	8.28E-05	3.70E-02	-0.306	1.90E-03	2.87E-01
**HBG2**	11:5274419-5667019	protein_coding	39577	0.327	9.13E-05	3.93E-02	0.370	2.78E-05	**5.59E-02**
SKIL	3:170075465-170114623	protein_coding	1570	-0.252	9.53E-05	3.96E-02	-0.203	5.29E-03	3.45E-01
GPT2	16:46918289-46965209	protein_coding	100	-0.321	1.21E-04	4.87E-02	-0.281	1.32E-03	2.48E-01
STRBP	9:125871778-126030855	protein_coding	1034	-0.337	1.38E-04	5.37E-02	-0.282	2.84E-03	3.16E-01
ZNF445	3:44481261-44519162	protein_coding	2182	-0.223	1.46E-04	5.52E-02	-0.242	4.21E-04	1.69E-01
RASGRF1	15:79252288-79383115	protein_coding	84	-0.220	1.72E-04	6.30E-02	-0.196	2.89E-03	3.16E-01
FIGNL1	7:50511830-50518088	protein_coding	234	-0.351	1.80E-04	6.39E-02	-0.360	2.40E-04	1.44E-01
PRKX	X:3522410-3631649	protein_coding	2556	-0.252	1.89E-04	6.52E-02	-0.206	7.62E-03	3.71E-01
CLNK	4:10488018-10686489	protein_coding	96	-0.288	2.03E-04	6.70E-02	-0.237	4.80E-03	3.38E-01
DDX6	11:118620033-118661858	protein_coding	10805	-0.183	2.17E-04	6.70E-02	-0.135	2.07E-02	4.49E-01
DESI2	1:244816236-244872335	protein_coding	945	-0.265	2.18E-04	6.70E-02	-0.292	3.43E-04	1.50E-01
NPDC1	9:139933921-139940655	protein_coding	146	0.344	2.19E-04	6.70E-02	0.355	2.36E-04	1.44E-01
NXF1	11:62559594-62573774	protein_coding	7228	0.164	2.22E-04	6.70E-02	0.197	2.06E-04	1.44E-01
RNF113A	X:119004496-119005791	protein_coding	297	0.335	2.60E-04	7.37E-02	0.363	1.92E-04	1.44E-01
RARS	5:167913449-167946304	protein_coding	1359	-0.274	2.65E-04	7.37E-02	-0.160	5.52E-02	5.36E-01
RHOBTB2	8:22844929-22877712	protein_coding	558	-0.335	2.66E-04	7.37E-02	-0.335	5.42E-04	1.82E-01
UGGT1	2:128848773-128953251	protein_coding	4889	-0.181	2.69E-04	7.37E-02	-0.185	1.51E-03	2.48E-01
ISG15	1:948802-949920	protein_coding	635	0.336	2.81E-04	7.52E-02	0.349	2.60E-04	1.44E-01
TIPARP	3:156391023-156424559	protein_coding	938	-0.261	2.87E-04	7.52E-02	-0.278	4.96E-04	1.82E-01
KSR2	12:117890816-118406788	protein_coding	98	-0.153	2.95E-04	7.57E-02	-0.173	3.74E-04	1.55E-01
ATP6V0D1	16:67471916-67515140	protein_coding	8907	0.203	3.21E-04	8.07E-02	0.188	4.15E-03	3.38E-01
FBXO11	2:48016454-48132932	protein_coding	1609	-0.228	3.35E-04	8.11E-02	-0.255	5.24E-04	1.82E-01
ZNF274	19:58694395-58724928	protein_coding	840	-0.287	3.36E-04	8.11E-02	-0.218	1.36E-02	4.15E-01
MCOLN1	19:7587511-7598895	protein_coding	1777	0.321	3.48E-04	8.23E-02	0.279	3.50E-03	3.22E-01
DDX24	14:94517265-94547591	protein_coding	2085	-0.266	3.55E-04	8.24E-02	-0.305	2.74E-04	1.44E-01
PEX26	22:18560688-18613905	protein_coding	1308	-0.265	3.69E-04	8.35E-02	-0.263	1.54E-03	2.48E-01
TBC1D23	3:99979843-100044095	protein_coding	1794	-0.218	3.74E-04	8.35E-02	-0.173	1.49E-02	4.21E-01
WNT3	17:44839871-44910520	protein_coding	137	-0.237	3.94E-04	8.58E-02	-0.240	9.18E-04	2.26E-01

RAB30	11:82684174-82782965	protein_coding	804	-0.313	4.01E-04	8.58E-02	-0.257	6.75E-03	3.71E-01
ODF3B	22:50968138-50971009	protein_coding	533	0.334	4.06E-04	8.58E-02	0.358	2.74E-04	1.44E-01
CDK2AP2	11:67273967-67276120	protein_coding	771	0.320	4.25E-04	8.72E-02	0.313	1.19E-03	2.43E-01
CDKN2D	19:10677137-10679735	protein_coding	4162	0.276	4.31E-04	8.72E-02	0.284	1.04E-03	2.36E-01
SLC6A16	19:49792894-49828482	protein_coding	611	-0.324	4.34E-04	8.72E-02	-0.238	1.45E-02	4.21E-01
MOAP1	14:93648540-93651273	protein_coding	427	0.320	4.45E-04	8.81E-02	0.292	2.59E-03	3.10E-01
IRF4	6:391738-411447	protein_coding	1629	-0.296	4.66E-04	9.07E-02	-0.260	4.53E-03	3.38E-01
S100PBP	1:33282367-33324476	protein_coding	2679	-0.231	4.81E-04	9.13E-02	-0.155	4.07E-02	5.05E-01
GPR15	3:98250742-98251960	protein_coding	181	0.282	4.84E-04	9.13E-02	0.266	2.04E-03	2.91E-01
**DNAH7**	2:196602426-196933536	protein_coding	151	-0.326	5.15E-04	9.39E-02	-0.392	5.33E-05	**7.15E-02**
TCF20	22:42556018-42739622	protein_coding	4310	-0.206	5.21E-04	9.39E-02	-0.147	3.31E-02	4.88E-01
EIF2S3L	12:10658200-10675734	protein_coding	393	-0.273	5.22E-04	9.39E-02	-0.227	9.18E-03	3.84E-01
PSMB6	17:4699438-4701790	protein_coding	615	0.315	5.61E-04	9.95E-02	0.271	5.23E-03	3.45E-01
LINC00452	13:114586639-114588308	lincRNA	14	-1.329	1.44E-05	4.53E-02	-1.268	8.05E-05	2.63E-01
RP11-481J2.2	16:58455229-58496374	lincRNA	10	-1.214	6.33E-05	9.97E-02	-1.116	4.67E-04	3.58E-01

#9 clinical covariates are DBP, TC, HDL, smoking, diabetes and treatments for hypertension, lipid, diabetes and aspirin, which are diifferent across MI, high CAC and controls (P<0.05, see Table 1).
Model 1: adjusted for sex, batch, and two SVs (results were shown in Table 2)

Model 2: adjsuted for additional 9 covariates (9 genes remained significant at FDR<0.1

Supplemental Table 7. GSEA pathway analysis and leading-edge genes (FDR<0.25 including 215 gene sets are upregulated in phenotype MI, and 8 gene sets are upregulated in controls)

NAME in MSigDB	SIZE	NES	NOM p-val	FDR q-val
BOWIE_RESPONSE_TO_EXTRACELLULAR_MATRIX	17	0.834	0.000	0.000
DAZARD_UV_RESPONSE_CLUSTER_G4	15	0.811	0.000	0.000
BOWIE_RESPONSE_TO_TAMOXIFEN	18	0.800	0.000	0.000
ZHANG_INTERFERON_RESPONSE	22	0.760	0.000	0.001
BENNETT_SYSTEMIC_LUPUS_ERYTHEMATOSUS	30	0.730	0.000	0.002
ZHANG_ANTIVIRAL_RESPONSE_TO_RIBAVIRIN_UP	20	0.725	0.000	0.002
CREIGHTON_AKT1_SIGNALING_VIA_MTOR_DN	20	0.687	0.000	0.010
UROSEVIC_RESPONSE_TO_IMIQUIMOD	20	0.688	0.000	0.011
STAMBOLSKY_TARGETS_OF_MUTATED_TP53_DN	38	0.675	0.000	0.012
CHIBA_RESPONSE_TO_TSA_UP	29	0.677	0.000	0.012
DAZARD_UV_RESPONSE_CLUSTER_G24	15	0.681	0.002	0.012
MOSERLE_IFNA_RESPONSE	29	0.671	0.000	0.014
EINAV_INTERFERON_SIGNATURE_IN_CANCER	26	0.663	0.000	0.016
GALE_APL_WITH_FLT3_MUTATED_DN	16	0.653	0.000	0.019
LIANG_SILENCED_BY_METHYLATION_2	32	0.655	0.000	0.020
LIANG_HEMATOPOIESIS_STEM_CELL_NUMBER_QTL	15	0.653	0.006	0.020
JOSEPH_RESPONSE_TO_SODIUM_BUTYRATE_UP	26	0.646	0.000	0.021
REACTOME_TRANSFERRIN_ENDOCYTOSIS_AND_RECYCLING	19	0.647	0.000	0.021
RASHI_NFKB1_TARGETS	18	0.648	0.000	0.022
CAVARD_LIVER_CANCER_MALIGNANT_VS_BENIGN	16	0.640	0.002	0.024
DAUER_STAT3_TARGETS_DN	46	0.633	0.000	0.027
HARRIS_BRAIN_CANCER_PROGENITORS	15	0.633	0.004	0.028
KRASNOSELSKAYA_ILF3_TARGETS_UP	28	0.633	0.000	0.029
RADAEVA_RESPONSE_TO_IFNA1_UP	47	0.627	0.000	0.031
KIM_LRRC3B_TARGETS	28	0.627	0.000	0.032

SUH_COEXPRESSED_WITH_ID1_AND_ID2_UP	16	0.624	0.006	0.032
NOJIMA_SFRP2_TARGETS_DN	18	0.621	0.002	0.034
XU_HGF_TARGETS_INDUCED_BY_AKT1_6HR	16	0.610	0.008	0.040
BROWNE_INTERFERON_RESPONSIVE_GENES	63	0.610	0.000	0.040
REACTOME_INTERFERON_ALPHA_BETA_SIGNALING	46	0.611	0.000	0.041
GRATIAS_RETINOBLASTOMA_16Q24	15	0.611	0.008	0.041
KANG_CISPLATIN_RESISTANCE_UP	16	0.612	0.000	0.041
WELCH_GATA1_TARGETS	18	0.614	0.000	0.042
OUYANG_PROSTATE_CANCER_PROGRESSION_DN	16	0.612	0.004	0.042
REACTOME_INSULIN_RECEPTOR_RECYCLING	17	0.602	0.000	0.049
AMUNDSON_GAMMA_RADIATION_RESPONSE	23	0.598	0.002	0.052
GNATENKO_PLATELET_SIGNATURE	43	0.592	0.000	0.057
PID_PRL_SIGNALING_EVENTS_PATHWAY	20	0.592	0.000	0.057
ZUCCHI_METASTASIS_DN	28	0.592	0.000	0.059
GENTILE_UV_LOW_DOSE_UP	22	0.586	0.002	0.059
VARELA_ZMPSTE24_TARGETS_UP	32	0.593	0.000	0.060
REACTOME_LATENT_INFECTION_OF_HOMO_SAPIENS_WITH_MYCOBACTERIUM_TUBERCULOSIS	23	0.585	0.004	0.060
XU_AKT1_TARGETS_6HR	25	0.586	0.002	0.061
NIKOLSKY_BREAST_CANCER_16P13_AMPLICON	71	0.584	0.000	0.061
HECKER_IFNB1_TARGETS	83	0.587	0.000	0.062
GOLUB_ALL_VS_AML_DN	21	0.588	0.000	0.062
VALK_AML_CLUSTER_7	25	0.586	0.000	0.062
DAZARD_UV_RESPONSE_CLUSTER_G2	18	0.581	0.002	0.064
SEITZ_NEOPLASTIC_TRANSFORMATION_BY_8P_DELETION_UP	47	0.577	0.000	0.067
SAGIV_CD24_TARGETS_DN	30	0.578	0.000	0.067
DIRMEIER_LMP1_RESPONSE_LATE_DN	28	0.578	0.002	0.068
DELLA_RESPONSE_TO_TSA_AND_BUTYRATE	19	0.576	0.010	0.068
KIM_HYPOXIA	19	0.573	0.008	0.070
VILIMAS_NOTCH1_TARGETS_DN	16	0.574	0.008	0.070
IIZUKA_LIVER_CANCER_PROGRESSION_L0_L1_DN	21	0.573	0.002	0.071
REACTOME_PYRIMIDINE_METABOLISM	19	0.570	0.004	0.074

CROMER_TUMORIGENESIS_UP	22	0.563	0.009	0.084
CREIGHTON_AKT1_SIGNALING_VIA_MTOR_UP	30	0.564	0.000	0.084
NATSUME_RESPONSE_TO_INTERFERON_BETA_DN	46	0.561	0.000	0.084
REACTOME_RESPIRATORY_ELECTRON_TRANSPORT	61	0.560	0.000	0.084
WENG_POR_TARGETS_LIVER_UP	28	0.561	0.000	0.085
MOOTHA_VOXPHOS	82	0.559	0.000	0.085
GESERICK_TERT_TARGETS_DN	17	0.559	0.012	0.086
MAHADEVAN_RESPONSE_TO_MP470_DN	15	0.558	0.035	0.086
LEIN_ASTROCYTE_MARKERS	19	0.561	0.006	0.087
CHIANG_LIVER_CANCER_SUBCLASS_INTERFERON_UP	17	0.555	0.016	0.091
LU_TUMOR_VASCULATURE_UP	15	0.552	0.019	0.095
SATO_SILENCED_BY_METHYLATION_IN_PANCREATIC_CANCER_2	22	0.552	0.008	0.096
MARCHINI_TRABECTEDIN_RESISTANCE_UP	17	0.549	0.010	0.101
WESTON_VEGFA_TARGETS_6HR	23	0.548	0.008	0.102
MACLACHLAN_BRCA1_TARGETS_UP	18	0.544	0.014	0.106
SCHURINGA_STAT5A_TARGETS_UP	16	0.545	0.022	0.106
REACTOME_IRON_UPTAKE_AND_TRANSPORT	29	0.545	0.000	0.107
INGA_TP53_TARGETS	15	0.542	0.035	0.110
REACTOME_BASIGIN_INTERACTIONS	16	0.541	0.014	0.112
TAKAO_RESPONSE_TO_UVB_RADIATION_UP	82	0.540	0.000	0.113
MATZUK_CENTRAL_FOR_FEMALE_FERTILITY	15	0.538	0.036	0.114
ZHAN_MULTIPLE_MYELOMA_LB_DN	34	0.538	0.000	0.115
ZHAN_V1_LATE_DIFFERENTIATION_GENES_UP	30	0.538	0.002	0.115
BAUS_TFF2_TARGETS_UP	22	0.532	0.010	0.118
URS_ADIPOCYTE_DIFFERENTIATION_DN	15	0.532	0.041	0.118
GROSS_ELK3_TARGETS_DN	24	0.533	0.013	0.119
DER_IFN_ALPHA_RESPONSE_UP	67	0.534	0.000	0.119
MIZUSHIMA_AUTOPHAGOSOME_FORMATION	18	0.535	0.029	0.120
ZHU_CMV_8_HR_UP	40	0.533	0.000	0.120
BECKER_TAMOXIFEN_RESISTANCE_UP	33	0.533	0.000	0.120
VALK_AML_CLUSTER_5	33	0.535	0.002	0.121

PID_ARF_3PATHWAY	19	0.533	0.014	0.121
REACTOME_RIP_MEDIATED_NFKB_ACTIVATION_VIA_DAI	16	0.529	0.030	0.124
HOUSTIS_ROS	30	0.528	0.000	0.124
GERHOLD_ADIPOGENESIS_UP	36	0.528	0.000	0.125
FARMER_BREAST_CANCER_CLUSTER_1	39	0.526	0.002	0.130
REACTOME_RESPIRATORY_ELECTRON_TRANSPORT_ATP_SYNTHESIS_BY_CHEMIOSMOTIC_COUPLING_AND_HEAT_PRODUCTION_BY_UNCOUPLING_PROTEINS_	76	0.524	0.000	0.135
KEGG_OXIDATIVE_PHOSPHORYLATION	100	0.522	0.000	0.136
SANA_TNF_SIGNALING_UP	57	0.522	0.000	0.137
JECHLINGER_EPITHELIAL_TO_MESENCHYMAL_TRANSITION_UP	44	0.521	0.000	0.138
KIM_ALL_DISORDERS_CALB1_CORR_DN	21	0.519	0.000	0.138
OKAWA_NEUROBLASTOMA_1P36_31_DELETION	17	0.521	0.042	0.139
MAGRANGEAS_MULTIPLE_MYELOMA_IGG_VS_IGA_DN	19	0.520	0.034	0.139
KEGG_FRUCTOSE_AND_MANNOSE_METABOLISM	28	0.519	0.004	0.139
VERRECCHIA_RESPONSE_TO_TGFB1_C2	16	0.518	0.040	0.140
VALK_AML_CLUSTER_8	25	0.516	0.002	0.145
WANG_CLASSIC_ADIPOGENIC_TARGETS_OF_PPARG	17	0.515	0.037	0.148
KIM_PTEN_TARGETS_UP	15	0.514	0.052	0.150
GILDEA_METASTASIS	18	0.512	0.042	0.150
SHIN_B_CELL_LYMPHOMA_CLUSTER_3	23	0.513	0.006	0.150
OHGUCHI_LIVER_HNF4A_TARGETS_UP	25	0.512	0.008	0.151
CHANDRAN_METASTASIS_TOP50_DN	39	0.510	0.000	0.153
REACTOME_CHOLESTEROL_BIOSYNTHESIS	19	0.509	0.019	0.153
AMIT_EGF_RESPONSE_60_MCF10A	25	0.510	0.014	0.153
FUJII_YBX1_TARGETS_UP	28	0.510	0.002	0.154
SEMENZA_HIF1_TARGETS	25	0.508	0.012	0.155
KEGG_REGULATION_OF_AUTOPHAGY	19	0.507	0.016	0.155
NAKAMURA_ADIPOGENESIS_LATE_DN	20	0.507	0.025	0.156
HAHTOLA_MYCOSIS_FUNGOIDES_UP	17	0.508	0.045	0.157
REACTOME_SEMA4D_INDUCED_CELL_MIGRATION_AND_GROWTH_CONE_COLLAPSE	21	0.506	0.016	0.158
MARTINEZ_RESPONSE_TO_TRABECTEDIN_UP	56	0.503	0.002	0.166

SCHLESINGER_METHYLATED_DE_NOVO_IN_CANCER	27	0.501	0.004	0.171
PID_TOLL_ENDOGENOUS_PATHWAY	21	0.491	0.024	0.174
NADLER_OBESITY_UP	48	0.500	0.000	0.175
KEGG_PARKINSONS_DISEASE	97	0.491	0.000	0.175
BARRIER_COLON_CANCER_RECURRENCE_DN	17	0.500	0.062	0.175
CHIBA_RESPONSE_TO_TSA	39	0.488	0.002	0.175
WUNDER_INFLAMMATORY_RESPONSE_AND_CHOLESTEROL_UP	44	0.491	0.000	0.176
WANG_METASTASIS_OF_BREAST_CANCER_ESR1_UP	19	0.488	0.056	0.176
SATO_SILENCED_BY_DEACETYLATION_IN_PANCREATIC_CANCER	23	0.488	0.023	0.176
TAVOR_CEBPA_TARGETS_DN	26	0.498	0.014	0.176
SAKAI_CHRONIC_HEPATITIS_VS_LIVER_CANCER_DN	21	0.491	0.031	0.176
ABE_VEGFA_TARGETS_2HR	21	0.489	0.054	0.176
REACTOME_G_PROTEIN_BETA_GAMMA_SIGNALLING	21	0.492	0.046	0.177
INGRAM_SHH_TARGETS_DN	39	0.490	0.000	0.177
REACTOME_AMYLOIDS	43	0.492	0.002	0.177
MOOTHA_TCA	16	0.498	0.064	0.177
KASLER_HDAC7_TARGETS_2_DN	22	0.497	0.008	0.178
BIOCARTA_CELLCYCLE_PATHWAY	16	0.489	0.080	0.178
GALINDO_IMMUNE_RESPONSE_TO_ENTEROTOXIN	74	0.489	0.000	0.178
BOYAULT_LIVER_CANCER_SUBCLASS_G3_DN	34	0.497	0.000	0.178
RHODES_UNDIFFERENTIATED_CANCER	52	0.492	0.000	0.178
REACTOME_ACTIVATION_OF_KAINATE_RECEPTORS_UPON_GLUTAMATE_BINDING	20	0.490	0.034	0.178
REACTOME_FORMATION_OF_TUBULIN_FOLDING_INTERMEDIATES_BY_CCT_TRIC	17	0.493	0.062	0.179
DASU_IL6_SIGNALING_UP	43	0.492	0.000	0.179
BANDRES_RESPONSE_TO_CARMUSTIN_MGMT_24HR_DN	16	0.497	0.063	0.179
LIANG_HEMATOPOIESIS_STEM_CELL_NUMBER_SMALL_VS_HUGE_DN	27	0.493	0.004	0.179
KEGG_DRUG_METABOLISM_OTHER_ENZYMES	23	0.494	0.014	0.179
WANG_BARRETTS_ESOPHAGUS_AND_ESOPHAGUS_CANCER_DN	19	0.496	0.035	0.180
WHITE_NEUROBLASTOMA_WITH_1P36.3_DELETION	16	0.492	0.054	0.180
SASAI_RESISTANCE_TO_NEOPLASTIC_TRANSFROMATION	31	0.495	0.014	0.180
GRAHAM_CML_QUIESCENT_VS_NORMAL_QUIESCENT_UP	64	0.494	0.000	0.180

REACTOME_PURINE_METABOLISM	27	0.496	0.010	0.181
ZHOU_TNF_SIGNALING_30MIN	46	0.494	0.000	0.181
SESTO_RESPONSE_TO_UV_C4	16	0.495	0.055	0.181
BIOCARTA_NFKB_PATHWAY	20	0.485	0.031	0.182
KIM_GLIS2_TARGETS_UP	49	0.485	0.002	0.182
BRACHAT_RESPONSE_TO_CAMPTOTHECIN_UP	22	0.485	0.025	0.182
GRADE_METASTASIS_DN	41	0.486	0.006	0.183
SCIBETTA_KDM5B_TARGETS_UP	17	0.484	0.053	0.184
LI_LUNG_CANCER	28	0.483	0.004	0.187
BOSCO_INTERFERON_INDUCED_ANTIVIRAL_MODULE	68	0.481	0.000	0.191
JOHNSTONE_PARVB_TARGETS_2_UP	114	0.481	0.000	0.191
KYNG_RESPONSE_TO_H2O2_VIA_ERCC6	16	0.479	0.085	0.197
KEGG_ARACHIDONIC_ACID_METABOLISM	27	0.478	0.010	0.200
KEGG_CARDIAC_MUSCLE_CONTRACTION	44	0.477	0.002	0.200
REACTOME_ER_PHAGOSOME_PATHWAY	57	0.477	0.000	0.201
PID_IL8_CXCR1_PATHWAY	25	0.476	0.016	0.201
LEONARD_HYPOXIA	38	0.477	0.002	0.202
LEE_LIVER_CANCER	15	0.475	0.084	0.204
REACTOME_AMINO_ACID_TRANSPORT_ACROSS_THE_PLASMA_MEMBRANE	19	0.474	0.048	0.208
LI_DCP2_BOUND_MRNA	82	0.474	0.000	0.208
HASLINGER_B_CLL_WITH_17P13_DELETION	20	0.473	0.070	0.211
PLASARI_TGFB1_SIGNALING_VIA_NFIC_10HR_DN	19	0.472	0.043	0.211
HALMOS_CEBPA_TARGETS_DN	21	0.472	0.045	0.211
HONMA_DOCETAXEL_RESISTANCE	33	0.472	0.008	0.211
FRIDMAN_SENESCENCE_UP	57	0.470	0.000	0.212
KONDO_COLON_CANCER_HCP_WITH_H3K27ME1	15	0.470	0.118	0.213
MARKEY_RB1_CHRONIC_LOF_DN	73	0.471	0.000	0.213
REACTOME_ANTIGEN_PROCESSING_CROSS_PRESENTATION	69	0.469	0.000	0.215
RICKMAN_METASTASIS_DN	194	0.468	0.000	0.216
KEGG_GLYCOSAMINOGLYCAN_DEGRADATION	17	0.468	0.067	0.217
ICHIBA_GRAFT_VERSUS_HOST_DISEASE_D7_UP	83	0.469	0.000	0.217

REACTOME_GLYCOSPHINGOLIPID_METABOLISM	26	0.467	0.030	0.218
ELVIDGE_HYPOXIA_BY_DMOG_DN	44	0.467	0.004	0.219
YAN_ESCAPE_FROM_ANOIKIS	16	0.467	0.102	0.220
CHEN_LVAD_SUPPORT_OF_FAILING_HEART_DN	22	0.466	0.045	0.221
PID_SYNDECAN_1_PATHWAY	22	0.465	0.044	0.223
NEMETH_INFLAMMATORY_RESPONSE_LPS_UP	82	0.464	0.000	0.223
NIKOLSKY_BREAST_CANCER_1Q21_AMPLICON	30	0.464	0.010	0.224
PARK_TRETINOIN_RESPONSE_AND_RARA_PLZF_FUSION	19	0.464	0.057	0.224
YAO_TEMPORAL_RESPONSE_TO_PROGESTERONE_CLUSTER_9	53	0.464	0.000	0.225
MISSIAGLIA_REGULATED_BY_METHYLATION_UP	99	0.463	0.000	0.226
REACTOME_REGULATION_OF_ORNITHINE_DECARBOXYLASE_ODC	46	0.462	0.004	0.227
REACTOME_NEF_MEDIATES_DOWN_MODULATION_OF_CELL_SURFACE_RECEPTORS_BY_RECRUITIN G_THEM_TO_CLATHRIN_ADAPTERS	18	0.462	0.039	0.227
SHIN_B_CELL_LYMPHOMA_CLUSTER_8	30	0.462	0.010	0.228
ENK_UV_RESPONSE_KERATINOCYTE_UP	402	0.462	0.000	0.229
CHIARADONNA_NEOPLASTIC_TRANSFORMATION_KRAS_CDC25_UP	43	0.461	0.002	0.229
WU_HBX_TARGETS_2_UP	22	0.460	0.050	0.229
CHANG_IMMORTALIZED_BY_HPV31_DN	44	0.461	0.004	0.229
REACTOME_EXTRACELLULAR_MATRIX_ORGANIZATION	30	0.460	0.016	0.229
BROWNE_HCMV_INFECTION_30MIN_UP	33	0.459	0.010	0.230
LEE_LIVER_CANCER_E2F1_UP	37	0.459	0.014	0.232
BEGUM_TARGETS_OF_PAX3_FOXO1_FUSION_DN	25	0.456	0.038	0.234
NAKAYAMA_SOFT_TISSUE_TUMORS_PCA1_UP	51	0.457	0.000	0.234
DORSAM_HOXA9_TARGETS_DN	28	0.457	0.025	0.234
HUNSBERGER_EXERCISE_REGULATED_GENES	17	0.457	0.092	0.235
REACTOME_CTNNB1_PHOSPHORYLATION_CASCADE	15	0.457	0.148	0.235
WIKMAN_ASBESTOS_LUNG_CANCER_DN	25	0.457	0.037	0.235
TAKEDA_TARGETS_OF_NUP98_HOXA9_FUSION_3D_DN	20	0.458	0.077	0.235
BURTON_ADIPOGENESIS_PEAK_AT_0HR	43	0.455	0.006	0.239
LANDIS_ERBB2_BREAST_PRENEOPLASTIC_DN	43	0.454	0.004	0.240
TONKS_TARGETS_OF_RUNX1_RUNX1T1_FUSION_SUSTAINDED_IN_ERYTHROCYTE_UP	31	0.453	0.019	0.243

CASTELLANO_NRAS_TARGETS_UP	43	0.453	0.006	0.243
DIRMEIER_LMP1_RESPONSE_EARLY	59	0.453	0.000	0.244
JISON_SICKLE_CELL_DISEASE_UP	168	0.451	0.000	0.246
MARCHINI_TRABECTEDIN_RESISTANCE_DN	35	0.452	0.010	0.246
PID_IL8_CXCR2_PATHWAY	31	0.452	0.011	0.247
SEITZ_NEOPLASTIC_TRANSFORMATION_BY_8P_DELETION_DN	16	0.451	0.136	0.247
MYLLYKANGAS_AMPLIFICATION_HOT_SPOT_23	17	-0.665	0.000	0.148
HUTTMANN_B_CLL_POOR_SURVIVAL_DN	52	-0.610	0.000	0.191
REACTOME_DOUBLE_STRAND_BREAK_REPAIR	21	-0.588	0.002	0.201
KEGG_ALANINE_ASPARTATE_AND_GLUTAMATE_METABOLISM	18	-0.592	0.004	0.204
CHEOK_RESPONSE_TO_MERCAPTOPURINE_AND_HD_MTX_DN	20	-0.601	0.002	0.212
IVANOVA_HEMATOPOIESIS_STEM_CELL_SHORT_TERM	18	-0.582	0.006	0.215
REACTOME_SIGNALING_BY_FGFR1_FUSION_MUTANTS	18	-0.613	0.002	0.220
REACTOME_SIGNALING_BY_FGFR1_MUTANTS	19	-0.594	0.000	0.221

Supplemental Table 8. 35 leading edge genes among the gene set of 72 genes on Affymetrix Exon-array data

NAME	PROBE	GENE SYMBOL	GENE_TITLE	RANK IN GENE LIST	RANK METRIC SCORE
row_31	TIPARP	TIPARP	TCDD-inducible poly(ADP-ribose) polymerase	13751	-0.884
row_32	TCF4	TCF4	transcription factor 4	13774	-0.891
row_33	RAD52	RAD52	RAD52 homolog (S. cerevisiae)	13776	-0.891
row_34	SH3PXD2A	SH3PXD2A	SH3 and PX domains 2A	13930	-0.936
row_35	ARHGAP27	ARHGAP27	Rho GTPase activating protein 27	14102	-0.973
row_36	MECP2	MECP2	methyl CpG binding protein 2 (Rett syndrome)	14147	-0.984
row_37	RAB30	RAB30	RAB30, member RAS oncogene family	14155	-0.985
row_38	RARS	RARS	arginyl-tRNA synthetase	14509	-1.07
row_39	ADAM28	ADAM28	ADAM metallopeptidase domain 28	14546	-1.08
row_40	S100PBP	S100PBP	S100P binding protein	14620	-1.1
row_41	**FOXP1**	FOXP1	forkhead box P1	14742	-1.13
row_42	FBXO11	FBXO11	F-box protein 11	14832	-1.16
row_43	FCER2	FCER2	Fc fragment of IgE, low affinity II, receptor for (CD23)	15121	-1.25
row_44	DDX6	DDX6	DEAD (Asp-Glu-Ala-Asp) box polypeptide 6	15456	-1.37
row_45	HNRNPR	null	null	15496	-1.38
row_46	DDX24	DDX24	DEAD (Asp-Glu-Ala-Asp) box polypeptide 24	15607	-1.43
row_47	ATP6V0D1	ATP6V0D1	ATPase, H+ transporting, lysosomal 38kDa, V0 subunit d1	15612	-1.43
row_48	UQCRC1	UQCRC1	ubiquinol-cytochrome c reductase core protein I	15708	-1.47
row_49	TCF20	TCF20	transcription factor 20 (AR1)	15717	-1.47
row_50	CBLB	CBLB	Cas-Br-M (murine) ecotropic retroviral transforming sequence b	15744	-1.48
row_51	PRKX	PRKX	protein kinase, X-linked	15779	-1.5
row_52	CCDC141	null	null	16235	-1.7
row_53	**IRF4**	IRF4	interferon regulatory factor 4	16277	-1.72
row_54	REST	REST	RE1-silencing transcription factor	16373	-1.78
row_55	PAX5	PAX5	paired box gene 5 (B-cell lineage specific activator)	16568	-1.91
row_56	VPS13D	VPS13D	vacuolar protein sorting 13 homolog D (S. cerevisiae)	16642	-1.95

row_57	BACH2	BACH2	BTB and CNC homology 1, basic leucine zipper transcription factor 2	16791	-2.07
row_58	BLK	BLK	B lymphoid tyrosine kinase	16841	-2.11
row_59	PRKDC	PRKDC	protein kinase, DNA-activated, catalytic polypeptide	16869	-2.14
row_60	ITCH	ITCH	itchy homolog E3 ubiquitin protein ligase (mouse)	16948	-2.22
row_61	SKIL	SKIL	SKI-like	16980	-2.25
row_62	AFF3	AFF3	AF4/FMR2 family, member 3	17041	-2.33
row_63	UGGT1	null	null	17059	-2.35
row_64	FCRL2	FCRL2	Fc receptor-like 2	17145	-2.5
row_65	STRBP	STRBP	spermatid perinuclear RNA binding protein	17157	-2.53
